# Proinflammatory cytokines driving cardiotoxicity in COVID-19

**DOI:** 10.1093/cvr/cvad174

**Published:** 2023-12-02

**Authors:** Maria Colzani, Johannes Bargehr, Federica Mescia, Eleanor C Williams, Vincent Knight-Schrijver, Jonathan Lee, Charlotte Summers, Irina Mohorianu, Kenneth G C Smith, Paul A Lyons, Sanjay Sinha

**Affiliations:** Wellcome – MRC Cambridge Stem Cell Institute, Jeffrey Cheah Biomedical Centre, Cambridge Biomedical Campus, University of Cambridge, Puddicombe Way, CB2 0AW Cambridge, UK; Department of Medicine, School of Clinical Medicine, University of Cambridge, Cambridge Biomedical Campus, Addenbrooke's Hospital, Hills Rd, CB2 0SP Cambridge, UK; Wellcome – MRC Cambridge Stem Cell Institute, Jeffrey Cheah Biomedical Centre, Cambridge Biomedical Campus, University of Cambridge, Puddicombe Way, CB2 0AW Cambridge, UK; Department of Medicine, School of Clinical Medicine, University of Cambridge, Cambridge Biomedical Campus, Addenbrooke's Hospital, Hills Rd, CB2 0SP Cambridge, UK; Department of Medicine, School of Clinical Medicine, University of Cambridge, Cambridge Biomedical Campus, Addenbrooke's Hospital, Hills Rd, CB2 0SP Cambridge, UK; Cambridge Institute of Therapeutic Immunology and Infectious Disease, Jeffrey Cheah Biomedical Centre, Cambridge Biomedical Campus, Puddicombe Way, CB2 0AW Cambridge, UK; Wellcome – MRC Cambridge Stem Cell Institute, Jeffrey Cheah Biomedical Centre, Cambridge Biomedical Campus, University of Cambridge, Puddicombe Way, CB2 0AW Cambridge, UK; Wellcome – MRC Cambridge Stem Cell Institute, Jeffrey Cheah Biomedical Centre, Cambridge Biomedical Campus, University of Cambridge, Puddicombe Way, CB2 0AW Cambridge, UK; Department of Medicine, School of Clinical Medicine, University of Cambridge, Cambridge Biomedical Campus, Addenbrooke's Hospital, Hills Rd, CB2 0SP Cambridge, UK; Wellcome – MRC Cambridge Stem Cell Institute, Jeffrey Cheah Biomedical Centre, Cambridge Biomedical Campus, University of Cambridge, Puddicombe Way, CB2 0AW Cambridge, UK; Department of Medicine, School of Clinical Medicine, University of Cambridge, Cambridge Biomedical Campus, Addenbrooke's Hospital, Hills Rd, CB2 0SP Cambridge, UK; Department of Medicine, School of Clinical Medicine, University of Cambridge, Cambridge Biomedical Campus, Addenbrooke's Hospital, Hills Rd, CB2 0SP Cambridge, UK; Wolfson Lung Injury Unit, Heart and Lung Research Institute, Cambridge Biomedical Campus, Papworth Road, CB2 0BB Cambridge, UK; Wellcome – MRC Cambridge Stem Cell Institute, Jeffrey Cheah Biomedical Centre, Cambridge Biomedical Campus, University of Cambridge, Puddicombe Way, CB2 0AW Cambridge, UK; Department of Medicine, School of Clinical Medicine, University of Cambridge, Cambridge Biomedical Campus, Addenbrooke's Hospital, Hills Rd, CB2 0SP Cambridge, UK; Cambridge Institute of Therapeutic Immunology and Infectious Disease, Jeffrey Cheah Biomedical Centre, Cambridge Biomedical Campus, Puddicombe Way, CB2 0AW Cambridge, UK; Department of Medicine, School of Clinical Medicine, University of Cambridge, Cambridge Biomedical Campus, Addenbrooke's Hospital, Hills Rd, CB2 0SP Cambridge, UK; Cambridge Institute of Therapeutic Immunology and Infectious Disease, Jeffrey Cheah Biomedical Centre, Cambridge Biomedical Campus, Puddicombe Way, CB2 0AW Cambridge, UK; Wellcome – MRC Cambridge Stem Cell Institute, Jeffrey Cheah Biomedical Centre, Cambridge Biomedical Campus, University of Cambridge, Puddicombe Way, CB2 0AW Cambridge, UK; Department of Medicine, School of Clinical Medicine, University of Cambridge, Cambridge Biomedical Campus, Addenbrooke's Hospital, Hills Rd, CB2 0SP Cambridge, UK

**Keywords:** COVID-19, Cardiotoxicity, Inflammation, Stem cell derived cardiomyocytes

## Abstract

**Aims:**

Cardiac involvement is common in patients hospitalized with COVID-19 and correlates with an adverse disease trajectory. While cardiac injury has been attributed to direct viral cytotoxicity, serum-induced cardiotoxicity secondary to serological hyperinflammation constitutes a potentially amenable mechanism that remains largely unexplored.

**Methods and results:**

To investigate serological drivers of cardiotoxicity in COVID-19 we have established a robust bioassay that assessed the effects of serum from COVID-19 confirmed patients on human embryonic stem cell (hESC)-derived cardiomyocytes. We demonstrate that serum from COVID-19 positive patients significantly reduced cardiomyocyte viability independent of viral transduction, an effect that was also seen in non-COVID-19 acute respiratory distress syndrome (ARDS). Serum from patients with greater disease severity led to worse cardiomyocyte viability and this significantly correlated with levels of key inflammatory cytokines, including IL-6, TNF-α, IL1-β, IL-10, CRP, and neutrophil to lymphocyte ratio with a specific reduction of CD4^+^ and CD8^+^ cells. Combinatorial blockade of IL-6 and TNF-α partly rescued the phenotype and preserved cardiomyocyte viability and function. Bulk RNA sequencing of serum-treated cardiomyocytes elucidated specific pathways involved in the COVID-19 response impacting cardiomyocyte viability, structure, and function. The observed effects of serum-induced cytotoxicity were cell-type selective as serum exposure did not adversely affect microvascular endothelial cell viability but resulted in endothelial activation and a procoagulant state.

**Conclusion:**

These results provide direct evidence that inflammatory cytokines are at least in part responsible for the cardiovascular damage seen in COVID-19 and characterise the downstream activated pathways in human cardiomyocytes. The serum signature of patients with severe disease indicates possible targets for therapeutic intervention.

## Graphical Abstract

**Graphical Abstract:**
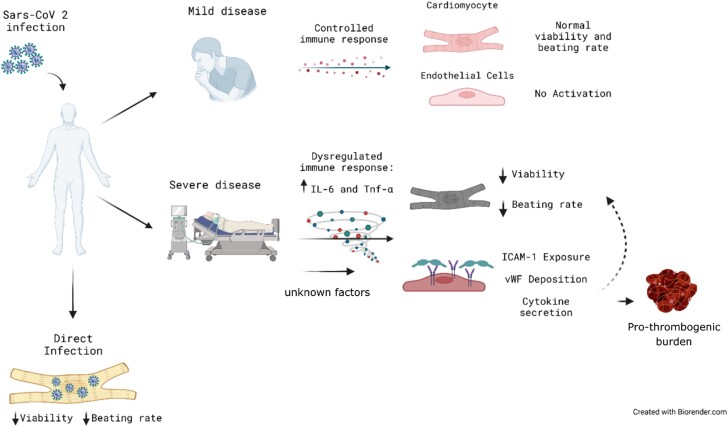

**Time of primary review: 18 days**

## 1. Introduction

Cardiac involvement is common in patients severely ill with COVID-19 and is associated with increased morbidity and mortality.^1–3^ The mechanism for myocardial injury in COVID-19 has been proposed to be at least in part due to a direct viral cytotoxic effect on cardiomyocytes. Pre-clinical data corroborate the notion that SARS-CoV-2 can enter and replicate within hiPSC-derived cardiomyocytes.^4–6^ Furthermore, co-expression of the ACE2 receptor and its accessory protease TMPRSS2 has been demonstrated in cardiomyocytes at the protein level in explanted human hearts, indicating that viral uptake is possible in principle.^7,8^

Nevertheless, post-mortem studies have not provided evidence for direct viral myocardial involvement in COVID-19. Investigating the presence of viral RNA in the myocardium of patients who died with COVID-19, Lindner *et al*. demonstrated viral RNA localized to interstitial cells but not cardiomyocytes with viral replication rarely encountered and a lack of histological evidence of viral myocarditis.^9^ One further study investigating myocardial biopsies in patients with suspected myocarditis, revealed a very low yield with only 5 out of 104 biopsies positive for SARS-CoV-2 and only 1 meeting the Dallas criteria for myocarditis.^10^ In line with this, Halushka *et al.* have estimated the prevalence of myocarditis in COVID-19 below 2%, examining 277 autopsied hearts across 22 publications of patients with confirmed COVID-19.^11^ These data suggest that direct viral myocardial involvement in COVID-19 is rare and may not be clinically relevant in the majority of cases.

At the same time, raised serological levels of cardiac troponin I (cTnI) reflect myocardial damage and independently predict a fatal outcome in COVID-19.^1,12–15^ Acute myocardial injury is a more adverse prognostic sign than other traditional risk factors, including age, diabetes or chronic obstructive pulmonary disease, making it a major determinant of a complicated disease trajectory with greater levels of serum Troponin signifying more severe myocardial damage.^16^ Given the significant cardiac involvement seen in the clinical syndrome but the limited evidence for a direct viral myocarditis, the mechanism of cardiac injury in COVID-19 remains poorly understood.

Alternatively, cardiac injury may result from excessive inflammatory cytokine production which is the main driver for the pathogenesis in COVID-19. This is corroborated by increased serum levels of the inflammatory cytokines interleukin (IL)-6, IL-10, and tumor necrosis factor alpha (TNF-α) in patients with COVID-19, which are associated with greater morbidity and mortality.^3,17^ Hence the aim of this study was to investigate whether a non-viral inflammatory mechanism was a possible cause of cardiac injury in patients with COVID-19.

## 2. Methods

### 2.1 Study design and participants

Study participants were prospectively enrolled between 31/3/2020 and 20/7/2020 at Addenbrooke’s Hospital (Cambridge, UK). A full description of the cohort is provided in Bergamaschi and colleagues.^18^ Briefly, all COVID-19 cases had a diagnosis confirmed with NAAT. Healthy controls were recruited among health care workers with negative SARS-CoV2 (Severe acute respiratory syndrome coronavirus 2) serology. Ethical approval was obtained from the East of England—Cambridge Central Research Ethics Committee (‘NIHR BioResource’ REC ref 17/EE/0025, and ‘Genetic variation AND Altered Leucocyte Function in health and disease—GANDALF’ REC ref 08/H0308/176). All participants provided informed consent. The study conforms to the principles outlined in the Declaration of Helsinki.

Serum samples were subsequently frozen down and thawed for the current study after selection of a subset of cases. All serum samples were confirmed negative for viral RNA on SARS-CoV2 RT-PCR before testing in the bioassay. SARS-CoV2 RNA positive samples were excluded from the study. Clinical characteristics were recorded, and study participants were assigned to the different groups based on COVID-19 severity:

1. Mild: health care workers recruited through the Addenbrooke’s staff screening programme,^16^ with mild COVID-19 symptoms and no need of hospitalization (*n* = 8).
2. Oxygen not required: hospitalized patients who did not receive any supplemental oxygen (*n* = 9).
3. Oxygen-non assisted: hospital patients who received supplemental oxygen using low flow nasal prongs, simple face mask, Venturi mask, or non-re-breather face mask (*n* = 9).
4. Assisted ventilation: hospital patients who received invasive ventilation (*n* = 13). Patients who received supplemental oxygen (but no ventilation) and deceased in hospital were also assigned to this group (*n* = 1 in this case series).
5. Healthy controls: Healthy controls were recruited among health care workers with negative SARS-CoV2 serology (*n* = 12).

For the first study time point samples were collected on average 14 days (±8.9 days) after symptom onset. For the follow-up time point samples were collected on average 43 days (±9 days) after symptom onset. No significant correlation was observed between the time of collection and cardiomyocyte viability.

All samples referred to as acute respiratory distress syndrome (ARDS) samples were obtained from mechanically ventilated adults who met the Berlin criteria for the diagnosis of ARDS, were admitted prior to the existence of SARS-CoV-2 and were receiving invasive mechanical ventilation (under REC reference 08/H0306/17).

### 2.2 Serology measurements:

High sensitivity C-reactive protein (CRP) was measured using the standard assay by the Core Biochemical Assay Laboratory (CBAL) at Cambridge University Hospitals NHS Foundation Trust.

IL-6, IL-10, IL-1β, TNF-α and IFN-γ were measured in serum from patients and HCs (healthy controls) by high sensitivity Base Kit HS Cytokine A Mag (cat# LHSCM000, R&D Systems/Biotechne) on a Luminex analyzer (Bio-Plex, Bio-Rad, UK) as standard clinical assay performed by the Clinical Immunology Laboratory at the Department of Biochemistry and Immunology, Addenbrooke’s Hospital Cambridge.

Complement components were measured in EDTA plasma from patients using commercially available enzyme-linked immunosorbent assays (ELISA) kits (HK354 (C3a), HK368 (C3c), HK328 (TCC), Hycult Biotech, Uden, the Netherlands) according to the manufacturer’s protocols.

### 2.3 Peripheral blood mononuclear cell preparation and flow immunophenotyping

Absolute count (cells/µL) of cell populations derived from peripheral blood mononuclear cells measured by flow cytometry was obtained from the NIHR CITIID COVID-19 Cohort database (https://www.covid19cellatlas.org/patient/citiid/).^18^

### 2.4 Cell culture and serum treatment

Cardiomyocytes were differentiated from H9 hESCs (human embryonic stem cells) and BOBC hiPSCs (human Induced pluripotent stem cells) as previously described.^19,20^ BOBC iPSCs (male) were a gift from Professor Ludovic Vallier. RUES-2 hESCs (female) were a gift from Professor Charles Murry and were cultured as previously described.^21^ RUES-2 and BOBC derived cardiomyocytes were generated using a protocol adapted from Burridge and colleagues^22^ (see Supplementary material online, *Figure S1*).

Following the onset of beating, cardiomyocytes were dissociated with TryPLE (Life Technologies) and plated onto Matrigel coated 96 well plates (10^5^ cells per well). 48 h after plating the media was then changed to chemically defined medium-bovine serum albumin (CDM-BSA) supplemented with 10% serum from the selected patients. This concentration was chosen based on the study from Kumar *et al.* which shows that serum from patients with septic shock can induce apoptosis in cardiomyocytes when used at this concentration.^23^ Serum containing media was refreshed after 48 h. Viability and contractility were measured at day 4 (i.e. after 4 days in total of incubation with serum).

Human microvascular endothelial cells [HMVEC, PromoCell (C-12281)] were a gift from Professor Nicholas Morell and were cultured according to the manufacturer’s instructions. Prior to serum treatment cells were seeded in 96 well plates at a density of 1.5 × 10^4^ cells per well in endothelial cell serum free media supplemented with 10% serum from selected patients. Media was refreshed after 48 h and cells were analysed on day 4 for viability and ICAM-1 membrane expression. Before von Willebrand factor (vWF) staining, serum containing media was removed and cells were cultured for an additional 2 days.

HL-60 cells were kindly gifted by Professor Brian Huntly and were cultured in RPMI 1640 (Gibco) supplemented with 10% fetal bovine serum (FBS) (Gibco) and Penicillin/Streptomycin.

### 2.5 Cytokine blocking and spike-in

For the cytokine blocking experiment cells were pre-incubated with either phosphate bufferred saline (PBS) or blocking antibodies (anti IL-6, cat. MAB206-SP, R&D 0.15 µg/mL and anti TNF-α, cat. AF-410-SP, R&D 10 µg/mL) for 4 h before the addition of the patients’ serum. Blocking antibodies or PBS were also added at the time of serum addition. Media containing serum and blocking antibodies was refreshed after 48 h and viability and contractility were measured on day 4. For the spike in experiment, IL-6 and TNF- α (Peprotech) were added to media supplemented with serum from control COVID-19 negative patients at the concentration of 100 and 150 pg/mL, respectively.

### 2.6 Cell viability assays

To assess cell viability PrestoBlue™ Cell Viability Reagent was added in a 1 in 10 dilution directly to the cardiomyocytes and incubated at 37°C for 3 h. Media was then removed and replaced with CDM BSA before further analysis. Fluorescence, which is a direct measurement of cell viability, was subsequently read at an excitation wavelength of 560 nm and an emission of 590 nm with absorbance monitored at 570 nm with a reference wavelength of 600 nm as per the manufacturer’s instructions. Viability data was obtained normalizing the fluorescence values to the values obtained in our control culture where cardiomyocytes were cultured in normal medium with no serum set as 1. Cell viability was assessed on fully differentiated cardiomyocytes depending on the differentiation protocol required for the respective cell line as specified in Supplementary material online, *F*igure *S1*. This was done because the absolute fluorescence reading might vary between experiments and plates and normalization to the control makes the results more consistent and comparable. The use of PrestoBlue™ as a cell viability assay has been validated previously.^24^

### 2.7 Calcium imaging

Calcium imaging was performed as previously described.^21^ In brief, on Day 4, Fluo-4 AM (5 µg/mL, Life Technologies) was added to the cells for 30 min at 37°C, Fluo-4 AM containing media was the removed and Tyrode’s buffer was added to the cells prior to imaging. Videos were recorded on an Axiovert inverted microscope (Zeiss) using Apple iPhone 8 and ILab microscope phone adapter. Videos were subsequently analysed with MATLAB R2021a.

### 2.8 RNA extraction, bulk mRNA sequencing, and RT-qPCR

RNA was extracted using GenElute Mammalian total RNA Miniprep Kit (SIGMA) according to the manufacturer’s instructions. RNA libraries were prepared using 200 ng of total RNA using NEBNext^®^ Poly(A) mRNA Magnetic Isolation Module and NEBNext^®^ Ultra™ II Directional RNA Library Prep Kit for Illumina^®^. Libraires were then sequenced on a Novaseq S4 XP lane.

Initial sample QC (quality control) was performed on the raw FASTQ files using FastQC v0.11.8 and summarized using multiQC, version 1.9. To limit technical variation due to uneven sequencing depths, we subsampled raw samples with sequencing depth >80 to 80 M reads^25^; the resulting range of sequencing depths was reduced to 69.9 M to 80.0 M reads. The samples were aligned to the H. sapiens genome (Ensemble GRCh38.p13) using STAR 2.7.6a (paired-end mode).^26^ Expression quantification was performed using featureCounts v2.0.0.^27^

The noise level across samples was assessed using noisyR,^28^ applied to the output count matrix. Using the noisyR output, a noise-filtered count matrix, normalized using quantile normalization,^29^ we identified the differentially expressed genes using edgeR.^30^ Differential expression (DE) was performed [i] using thresholds |log2FC|>0.5 and adjusted *P*-value (with Benjamini-Hochberg multiple testing correction) < 0.05 between all mild/control samples together and severe samples and [ii] using threshold |log2FC|>0.5 between all mild/control samples together and each severe sample separately, labelling each comparison ‘U’ (up), ‘D’ (down) or ‘S’ (straight or non-DE).^31^ Enrichment analysis on all separate DE transcripts and on genes labelled ‘U’ or ‘D’ in 4/5/6/all of the comparisons was performed using the gprofiler2 package^32^ with the background set as the set of expressed genes with abundance above the signal/noise threshold in at least one sample. The inference of Gene Regulatory Networks (GRNs), using DE genes as focal genes, was performed using GENIE3.^33^ The visualization, and community accessible overview were compiled using bulkAnalyseR.^34^ RNA sequencing data is available on the NCBI Gene Expression Omnibus (https://www.ncbi.nlm.nih.gov/geo/) GEO accession number GSE207473 and can be visualized at http://bioinf.stemcells.cam.ac.uk/shiny/sinha/MColzani_COVID19_Cardiotoxicity/

For RT-qPCR, 100 ng of RNA was retrotranscribed to complementary DNA (cDNA) using Maxima First Strand cDNA Synthesis Kit (Thermo scientific). RT-qPCR was performed using Fast SYBR^®^ Green Master Mix on a 7500 Real-Time PCR System using GAPDH (glyceraldehyde 3-phosphate dehydrogenase) as housekeeping gene. All primers genes (listed in the table below) were designed to span an intron-exon junction. MRNA expression relative to GAPDH was calculated using the Delta Ct method.

**Table:** 

Gene	Fw primer	Rev primer
hGAPDH	AACAGCCTCAAGATCATCAGC	GGATGATGTTCTGGAGAGCC
MYH7	ACTGCCGAGACCGAGTATG	GCGATCCTTGAGGTTGTAGAGC
TNNI3	TTTGACCTTCGAGGCAAGTTT	CCCGGTTTTCCTTCTCGGTG
MYL2	TACGTTCGGGAAATGCTGAC	TTCTCCGTGGGTGATGATG

### 2.9 Flow cytometry

HMVEC were harvested using TrypLE (Gibco), and subsequently fixed with 4% PFA for 20 min at room temperature. Subsequently cells were washed and then stained with anti ICAM-1 (intercellular adhesion molecule 1) antibody or IgG control (Milteny, 130–120–780 diluted 1:30) in PBS containing 2 mM EDTA for 30 min at room temperature. Following a washing step, cells were analysed on the Attune Cell Analyzer flow cytometer. FlowJo 10.8.1 was subsequently used to analyse the acquired data.

### 2.10 Immunocytochemistry

Immunocytochemistry was performed as previously described.^35^ Briefly, HMVEC were fixed with 4%PFA (paraformaldehyde, Thermo Fisher Scientific) for 1 h at room temperature, permeabilized for 15 min with PBS supplemented with 0.3% Triton-X (Sigma) and 0.5% BSA (Sigma), blocking was performed with PBS supplemented with 5% BSA and 0.1% Triton-X. Cells were subsequently stained for vWF overnight at 4C (NB600–586, Novus Biologicals diluted 1:300 in PBS with 0.5% BSA). Cells were washed with PBS and incubated with anti-Rabbit 568 (Invitrogen, diluted 1:500) and DAPI (4′,6-diamidino-2-phenylindole, Sigma, 1 µg/mL) in PBS with 0.5% BSA for 2 h. Cells were then washed with PBS prior to imaging. Images were acquired on a Leica SP5 confocal microscope and analysed using FIJI ImageJ software.

### 2.11 Cardiac troponin-T ELISA

To assess cardiomyocyte death by Troponin-T release in the media, cardiomyocytes were cultured in the presence of serum as described above. The presence of cardiac Troponin-T in the undiluted media was detected using Human Cardiac Troponin T ELISA Kit (ab223860, abcam) following the manufacturer’s instructions. Serum containing media not exposed to the cells was used as a control to ensure that the Troponin detected in the cell culture supernatant was of cardiomyocyte origin.

### 2.12 HMVEC immune cell adhesion assay

HMVECs were cultured in the presence of serum as described previously. On day 4, media was removed, and cells were washed with PBS and 2 × 10^4^ HL-60 cells previously labelled with CellTracker Red CMTPX (ThermoFisher) were added to each well. Cells were then incubated for 2 h at 37 C, media was then removed, cells were washed once with PBS to remove any unbound HL-60 cells, cells were then fixed with 4% PFA and imaged using a EVOS microscope (ThermoFisher) and HL-60 cell adhesion was quantified on ImageJ.

### 2.13 Statistical analysis

All experimental data specifically state the number of patients assessed for quantitative endpoint analysis. The normality of the distributions was assessed using the Shapiro-Wilk test where appropriate. Statistical testing was performed using an unpaired *t*-test for two-group comparisons and a one-way ANOVA with a post-hoc Tukey test was used for multiple-group comparison. Measuring two-sided significance, a *P* value of less than 0.05 was considered statistically significant. All analyses were performed in a blinded fashion using GraphPad Prism software. All results are expressed as mean ± s.d., encompassing at least 50% of the data, unless otherwise stated.

## 3. Results

### 3.1 COVID-19 patient serum has a direct cardiotoxic effect on cardiomyocytes in vitro

**Figure 1:**
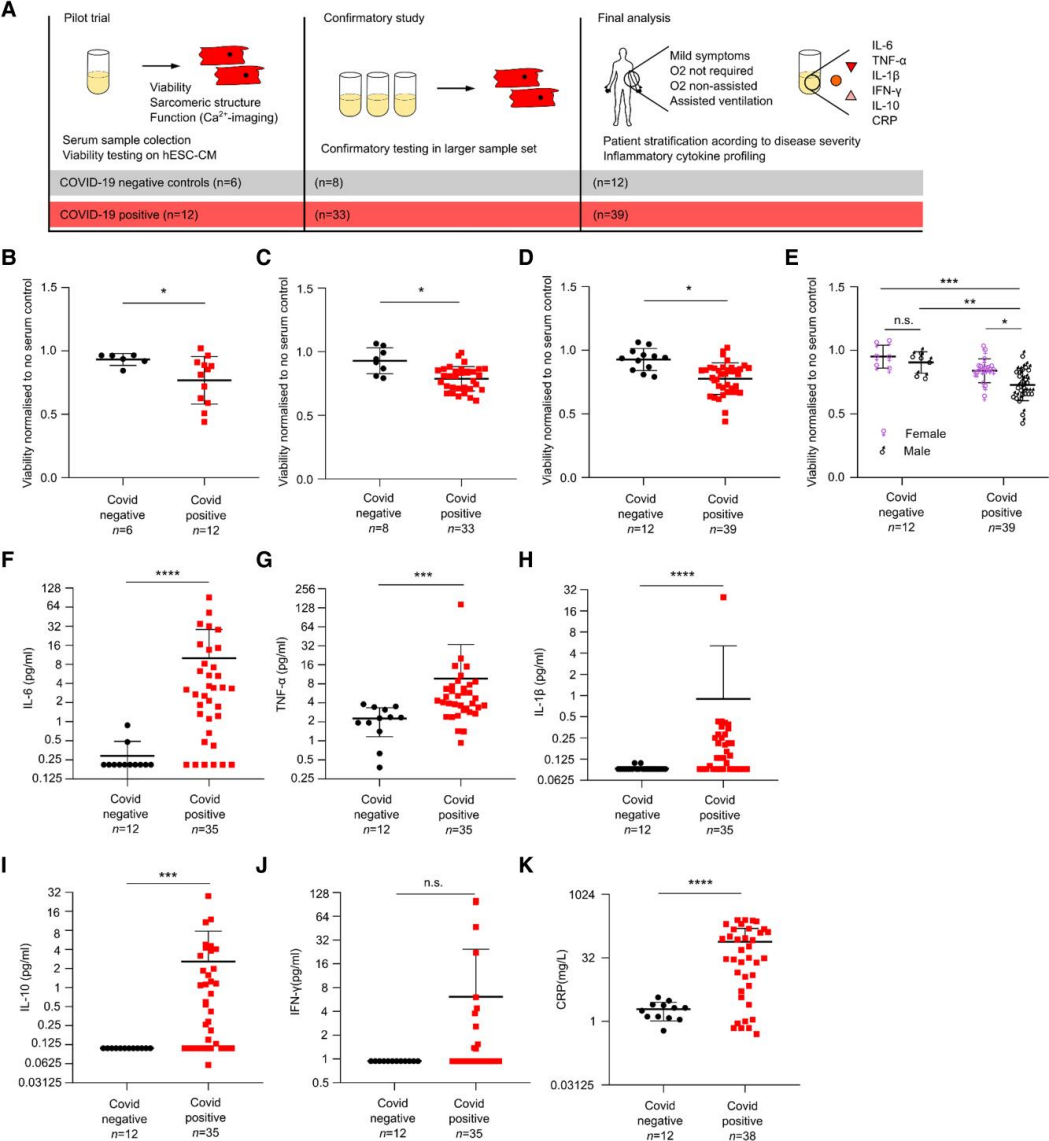
Serum cardiotoxicity in patients with COVID-19. (*A*) Schematic of study design. Assessing the effects of serum from patient with confirmed COVID-19 and negative controls on hESC-CM viability and subsequent cytokine profiling. Cardiomyocyte viability was assessed by change in PrestoBlue™ fluorescence and normalized to controls (hESC-CM without serum). (*B*) Viability of hESC-CM following exposure to serum of COVID-19 positive patients and controls, *n* = 6 and 12 patients. Viability data was obtained normalizing the fluorescence values to the values obtained in our control culture where cardiomyocytes were cultured in normal medium with no serum set as 1. (*C*) Results of larger confirmatory study. COVID-19 positive patients and negative controls, *n* = 8 and 33 patients. (*D*) Combination of study results from pilot trial and confirmatory study. COVID-19 positive patients and negative controls, *n* = 12 and 39 patients. The results from the 8 serum samples in common between the first and second study were plotted as the average of the two experiments. (*E*) Cardiomyocyte viability and sex distribution in COVID-19 positive patients and negative controls. (*F–K*) Luminex assay of patient serum samples, including IL-6 (*F*) TNF-α (*G*), IL1-β (*H*), IL-10 (*I*), IFN-γ (*J*), and high sensitivity CRP (*K*). Mean values; error bars represent s.d. Two-sided *P* values were calculated using an unpaired *t*-test unless otherwise stated. * *P* < 0.05, *** *P* < 0.001, **** *P* < 0.0001. Abbreviations: hESC-CM, human embryonic stem cell-derived cardiomyocytes; n.s., not significant.

First, we obtained serum samples from an initial cohort of patients with confirmed COVID-19 from the first COVID-19 wave (*n* = 12) and healthy probands (*n* = 6).^18^ Patient sera were obtained from inpatients who had tested positive for COVID-19 by naso-pharyngeal swab, while serum samples themselves were confirmed to be negative for SARS-CoV-2 by RT-PCR. Sera were subsequently tested on H9 human embryonic stem cell (hESC)-derived cardiomyocytes, which were generated as previously described.^19^ Cells were cultured for 4 days with media/serum change every other day. A schematic of the study design is shown in *Figure 1A* and the clinical characteristics of all patients and controls are provided in Supplementary material online, *Figure S2*.

We then assessed cardiomyocyte viability on day 4 of serum treatment by Presto Blue and found significantly decreased viability with COVID-19 + patient serum samples compared to healthy controls. This was paralleled by a reduction in beating rate as assessed by Ca^2+^-imaging (*Figure 1B*, see Supplementary material online, *Figure S3A*, Supplementary video file 1). We subsequently confirmed these findings in a larger cohort of COVID-19 + patients (*n* = 33) and healthy controls (*n* = 8). This second cohort included eight serum samples (two from control patients and six from COVID-19 + patients) that were previously tested in the first cohort (*Figure 1C*). Given, the robust reproducibility of our bioassay (variance of the first study 0.0098, variance of the second study 0.0065 with an F- Test *P*-value of 0.37), we have pooled these two cohorts for all further analyses (*Figure 1D*). We confirmed in a separate experiment that this effect is seen in cardiomyocytes derived from both male and female hPSC lines (see Supplementary material online, *Figure S3B*). In line with the clinical observation that men tend to have a more severe course of COVID-19 we demonstrated that male patient sera led to a significantly greater reduction in cardiomyocyte viability than female sera (*Figure 1E*). Luminex assays on patient sera demonstrated that as previously shown COVID-19 positive patients had significantly greater levels of IL-6, TNF-α, IL-1β, IL-10, and CRP (*Figure 1F–K*).^18^ Taken collectively, COVID-19 patient serum (which contains multiple proinflammatory cytokines) results in cardiomyocyte toxicity in our in vitro assay.

### 3.2 Serological cardiotoxicity correlates with COVID-19 disease severity

**Figure 2:**
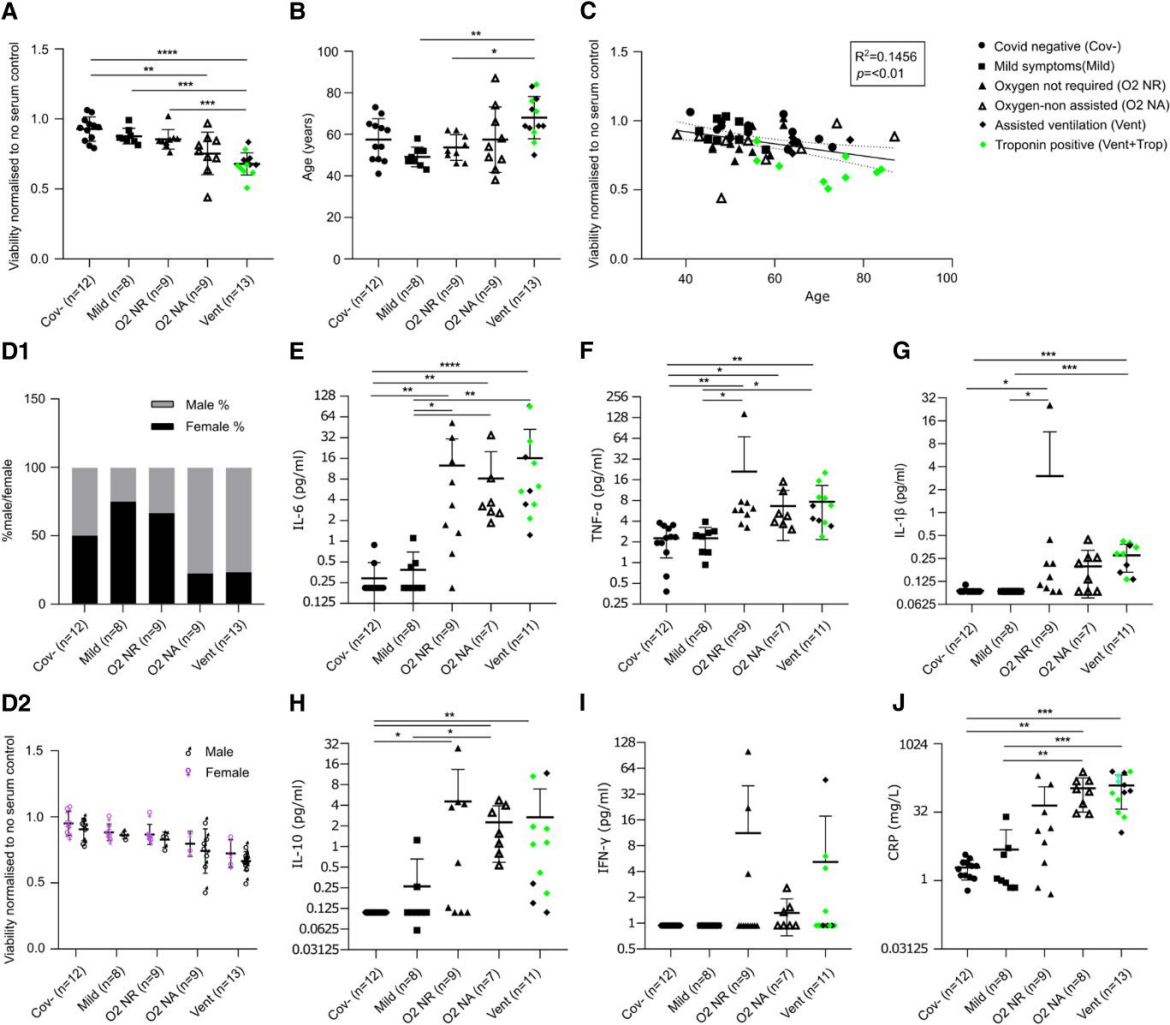
Cardiomyocyte viability correlates with COVID-19 disease severity and key pro-inflammatory cytokines. (*A*) hESC-CM viability data obtained normalizing the PrestoBlue™ fluorescence values to the values obtained in our control culture where cardiomyocytes were cultured in normal medium with no serum set as 1. (*B*) Age in patients stratified according to disease severity. (*C*) Correlation of age and hESC-CM viability. Cov-, mild, O_2_ NR, O_2_ NA, Vent, *n* = 12, 8, 9, 9, and 13 (*A–C*). (D1) Gender distribution amongst disease severity groups. Cov-, mild, O_2_ NR, O_2_ NA, Vent, *n* = 12, 8, 9, 9, and 13. (D2) hESC-CM viability amongst males and females stratified according to disease severity. (*E–J*) Luminex cytokine data across disease severity groups. Shown are IL-6 (*E*), TNF-α (*F*), IL1-β (*G*), IL-10 (*H*), IFN-γ (*I*), and CRP (*J*). Mean values; error bars represent s.d. Two-sided *P* values were calculated using a one-way ANOVA with post-hoc correction for multiple comparisons. * *P* < 0.05, ** *P* < 0.01, *** *P* < 0.001, **** *P* < 0.0001. Abbreviations: Cov-, COVID-19 negative; mild, mild symptoms only; O_2_ NR, oxygen not required; O_2_ NA, Oxygen-non assisted; vent, assisted ventilation; hESC-CM, human embryonic stem cell-derived cardiomyocytes.

We next stratified patients based on a previously published study according to disease severity, namely, COVID-19 negative (Cov-), mild symptoms (Mild), oxygen not required (O_2_ NR), Oxygen-non assisted (O_2_ NA) and assisted ventilation (Vent).^18^ Cardiomyocyte viability was significantly poorer and beating rate lower the greater the clinical disease burden but was preserved in COVID-19 negative controls. Cardiomyocyte death was also proven by increased levels of cardiac Troponin-T in the culture supernatant of cells cultured in the presence of a subset of severe patient sera compared to the controls (see Supplementary material online, *Figure S3*). Troponin positivity was exclusively encountered in serum from patients requiring assisted ventilation, highlighting its clinical role as a marker of disease severity (*Figure 2A*, see Supplementary material online, *Figure S3C*). We have confirmed in a separate experiment that serum treatment of hPSCs results in Troponin release in the media (see Supplementary material online, Figure S3D). Older patients were more likely to require assisted ventilation and age was significantly correlated with cardiomyocyte viability (*Figure 2B* and *C*). There were more females in the less severe and more males in the severe disease groups (14 males vs. 6 females in the O_2_ NA and Vent Groups), as expected, but there was no significant difference in viability between the genders within the disease groups (*Figure 2D*). To determine the drivers of cardiotoxicity, we carried out a Luminex assay and demonstrated that greater disease severity was paralleled by significantly greater levels of IL-6, TNF-α, IL-1β, IL-10, and CRP (*Figure 2E–J*).

**Figure 3:**
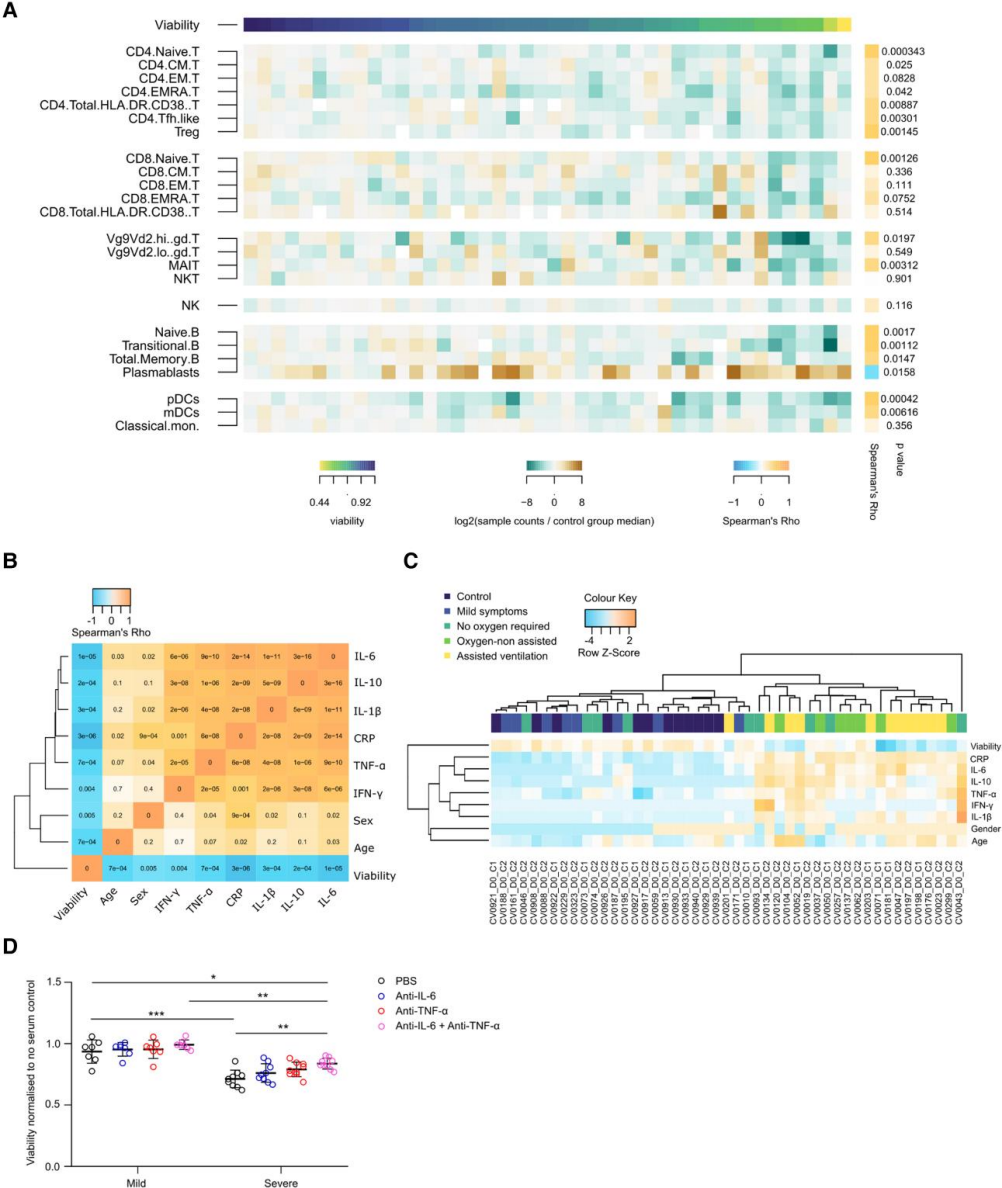
Serological profile of patients with COVID-19 and therapeutic blockage mitigating cardiotoxicity. (*A*) Heatmap showing the Log_2_ fold change in median absolute cell counts (bottom mid legend) in patients ordered according to cardiomyocyte viability (bottom left legend). The right column shows the Spearman’s Rho (bottom right legend) and associated *P* values. (*B*) Correlation matrix of key pro-inflammatory cytokines, gender, age, and hESC-CM viability. Top legend shows Spearman’s Rho. Values represent two-sided *P* values. (*C*) Hierarchical clustering of patients according to COVID-19 disease severity and pro-inflammatory cytokine response. Top legend represent raw Z score. (*D*) Combinatorial blockage of key pro-inflammatory cytokines increases hESC-CM viability. Viability data was obtained normalizing the fluorescence values to the values obtained in our control culture where cardiomyocytes were cultured in normal medium with no serum set as 1. Mean values; error bars represent s.d. Two-sided *P* values were calculated using a one-way ANOVA with post-hoc correction for multiple comparisons. * *P* < 0.05, ** *P* < 0.01, *** *P* < 0.001, **** *P* < 0.0001. Abbreviations: Cov-, COVID-19 negative; mild, mild symptoms only; O_2_ NR, oxygen not required; O_2_ NA, Oxygen-non assisted; vent, assisted ventilation; hESC-CM, human embryonic stem cell-derived cardiomyocytes.

Neutrophil counts were greater, and lymphocyte counts lower in patients requiring assisted ventilation compared to patients in whom supplemental O_2_ was not required (see Supplementary material online, *Figure S4A*–*C*). To elucidate the relationship between immune cell subsets and cardiomyocyte viability we performed immunophenotyping by flow cytometry of peripheral blood mononuclear cells. Cardiomyocyte viability correlated with a reduction of many CD4 + and CD8+ T-cells subsets and increase in plasmablast number, a feature associated with an increased disease severity, as recently reported by Bergamaschi *et al.* (*Figure 3A*). The role of serological components in causing cardiovascular damage in COVID-19 is further corroborated by a significant negative correlation of IL-6, TNF-α, IL-1β, IL-10, and CRP levels with cardiomyocyte viability (*Figure 3B*). Further analysis demonstrates that the majority of the pro-inflammatory cytokine response is seen in patients requiring oxygen or assisted ventilation (*Figure 3C*). There was no correlation between presence of cardiovascular complications and cardiomyocyte viability (see Supplementary material online, *Figure S4D*). Supplementary material online Supplementary material online, *Figures S5* and *S6* show the results of cohort 1 and 2, respectively.

In summary, serum cardiotoxicity correlates with pro-inflammatory cytokine expression and with clinical disease severity in COVID-19.

### 3.3 Therapeutic blockade of key pro-inflammatory cytokines ameliorates cardiotoxic damage

To confirm that cardiotoxic damage by the serum is driven by a pro-inflammatory cytokine response, we carried out a blocking study in which cardiomyocytes were cultured in the presence of patients’ serum as well as neutralizing antibodies against the top upregulated cytokines in severe patients, IL-6 and TNF-α or a combination of both. We found no effect on cardiomyocyte viability upon blockade of serum from patients with mild symptoms only. In contrast, we demonstrated a significant improvement in cardiomyocyte viability upon combinatorial blockade of IL-6 + TNF-α in sera of patients with severe disease, compared to vehicle control alone (*P* < 0.001) (*Figure 3D*). Spiking of control serum with commercially available human recombinant IL-6 and TNF-α did not reproduce these effects (see Supplementary material online, *Figure S7*). Finally, we followed up a subset of patients for 4 weeks and beyond and observed no significant difference in the effect of COVID-19 patient sera on cardiomyocyte viability compared to vehicle controls, which despite not reaching significance was accompanied by an overall trend towards the proinflammatory cytokine response resolving (see Supplementary material online, *Figure S8*).

Taken collectively, the pro-inflammatory response causing cardiotoxicity in COVID-19 is paralleled by a dysregulated immune response as seen by a reduction in CD4 + and CD8+ T-cells subsets in patients with severe disease. Combinatorial blockade of IL-6 and TNF-α in serum resulted in cardiomyocyte rescue, highlighting an opportunity for therapeutic intervention.

### 3.4 Serum cardiotoxicity is not unique to COVID-19 but the response is cell specific

**Figure 4:**
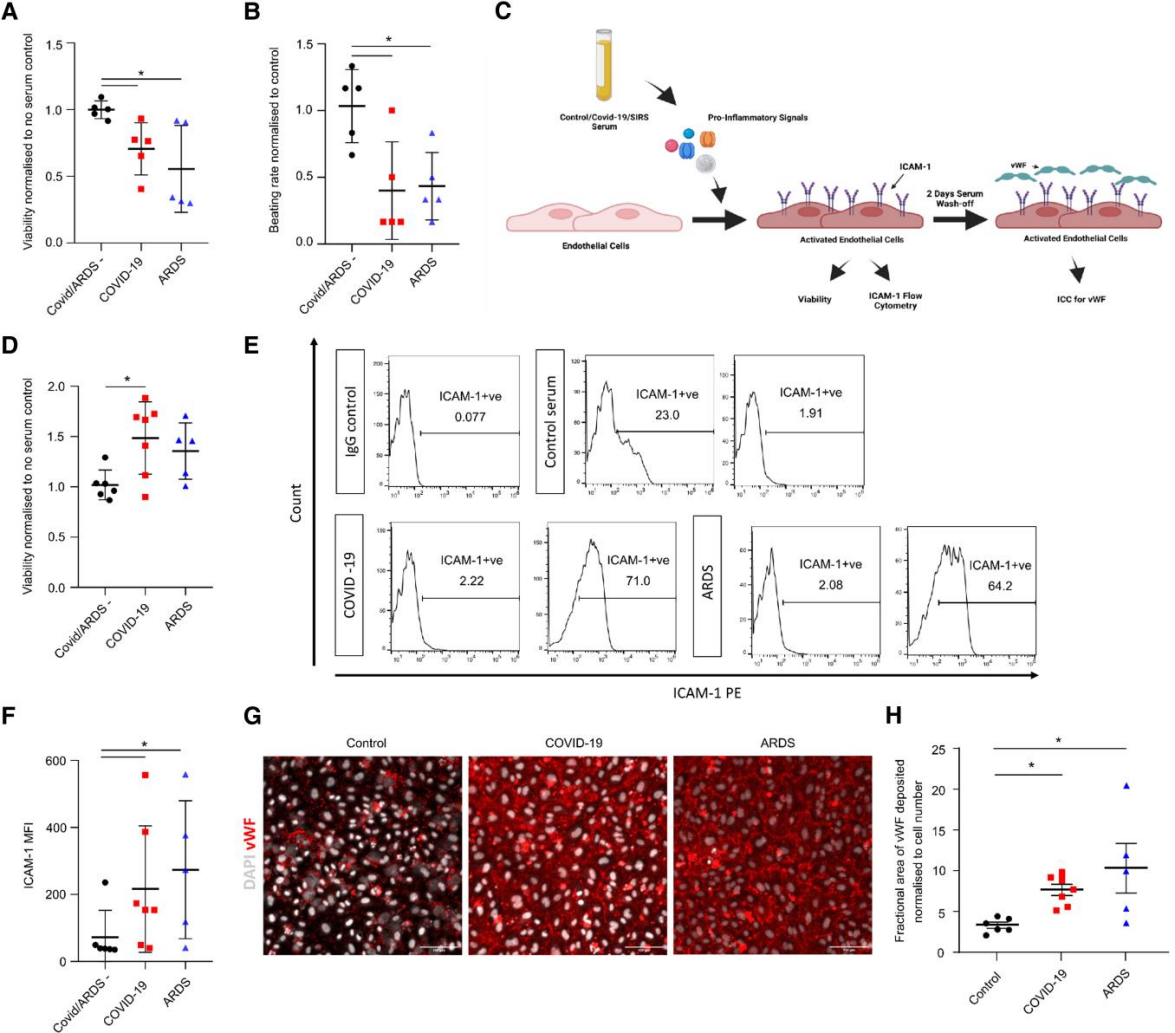
Specificity of COVID-19 serum toxicity and cell response: (*A*) viability of hESC-CM following exposure to serum from either healthy probands or patients with severe COVID-19 or patients with ARDS but without COVID-19. Viability data was obtained normalizing the PrestoBlue™ fluorescence values to the values obtained in our control culture where cardiomyocytes were cultured in normal medium with no serum set as 1. (*B*) Beating rate of hESC-CM (normalized to no serum controls) following exposure to serum from either healthy probands or patients with severe COVID-19 or patients with ARDS but without COVID-19. (*C*) Schematic illustrating the experimental set up using HMVECs to assess whether the COVID-19 serum response has cell-specific effects. (*D*) Viability of HMVEC (normalized to no serum control) following exposure to serum of control COVID-19 or ARDS patients and controls, *n* = 5, 7, and 5, respectively. (*E*) Representative flow cytometry histogram plots showing the percentage of ICAM-1 expressing cells following serum exposure. (*F*) ICAM-1 MFI of cells treated with serum of control COVID-19 or ARDS patients and controls, *n* = 5, 7, and 5, respectively. (*G*) Representative confocal images of HMVEC stained for vWF (red) and DAPI (white). Scale bar 100 µm. (*H*) Quantification of vWF deposition. *P < 0.05. Abbreviations: ARDS, acute respiratory distress syndrome.

To investigate whether the cardiotoxicity observed in response to patient serum was unique to COVID-19 we obtained samples from patients who were COVID-19 negative but suffered from ARDS requiring intubation and ventilation. Treatment with serum from patients with ARDS resulted in a significant reduction of cardiomyocyte viability and beating rate compared to treatment with serum from healthy probands (*Figure 4A* and *B*, see Supplementary material online, *Figure S3C*). There was no significant difference in cardiomyocyte viability or beating rate when comparing the effect of COVID-19 and ARDS samples.

To investigate whether the serum cytotoxicity observed in COVID-19 is unique to cardiomyocytes or also occurs in other cell types, we performed further experiments using microvascular endothelial cells since a common feature of severe forms of COVID-19 is severe vascular injury with micro- and macro-thrombotic events.^36^ Endothelial cells sparsely express ACE2 and mostly do not take up the virus.^36^ However, pericytes do strongly express ACE2,^37^ vascular organoids were shown to be permissive for SARS-CoV-2 infection^38^ and the virus uptake might be organ-specific.^39,40^ While, as for the cardiomyocytes we cannot rule out a direct effect of SARS-CoV-2 on endothelial cells, we wanted to assess whether COVID-19 serum exposure results in endothelial cell activation. HMVECs were exposed to severe COVID-19, ARDS and control sera. This resulted in a significant increase in endothelial cell viability (non-significant trend in ARDS group) possibly indicating endothelial cell activation (*Figure 4C* and *D*). A hallmark of endothelial cell activation is ICAM-1 membrane expression leading to immune cell adhesion and vWF deposition that can later trigger platelet activation. To investigate the activation state of endothelial cells we assessed ICAM-1 expression following serum exposure using flow cytometry. Despite some variability in the response, we observed an increase in the percentage of ICAM-1 expressing cells following treatment with severe COVID-19 or ARDS sera. Furthermore, this was mirrored by a significant increase in ICAM-1 mean fluorescence intensity (MFI) in the severe COVID-19 and ARDS cell cohorts compared to the controls (*Figure 4E* and *F*). Interestingly we also observed an increase in vWF deposition in the extracellular matrix by endothelial cells that had undergone treatment with COVID-19 or ARDS serum but not by those treated with control serum (*Figure 4G* and *H*). The pro-inflammatory activated state of the endothelial cells was further confirmed by increased adhesion of monocyte like cells (HL-60) onto HMVECs culture in the presence of severe COVID or ARDS sera compared to healthy ones (see Supplementary material online, *Figure S9*).

To conclude, these data demonstrate that serum-induced cytotoxicity is not unique to COVID-19 but it triggers a cell-specific response resulting in endothelial cell activation thereby creating a procoagulant state.

### 3.5 RNA sequencing analysis of serum treated cardiomyocytes

**Figure 5:**
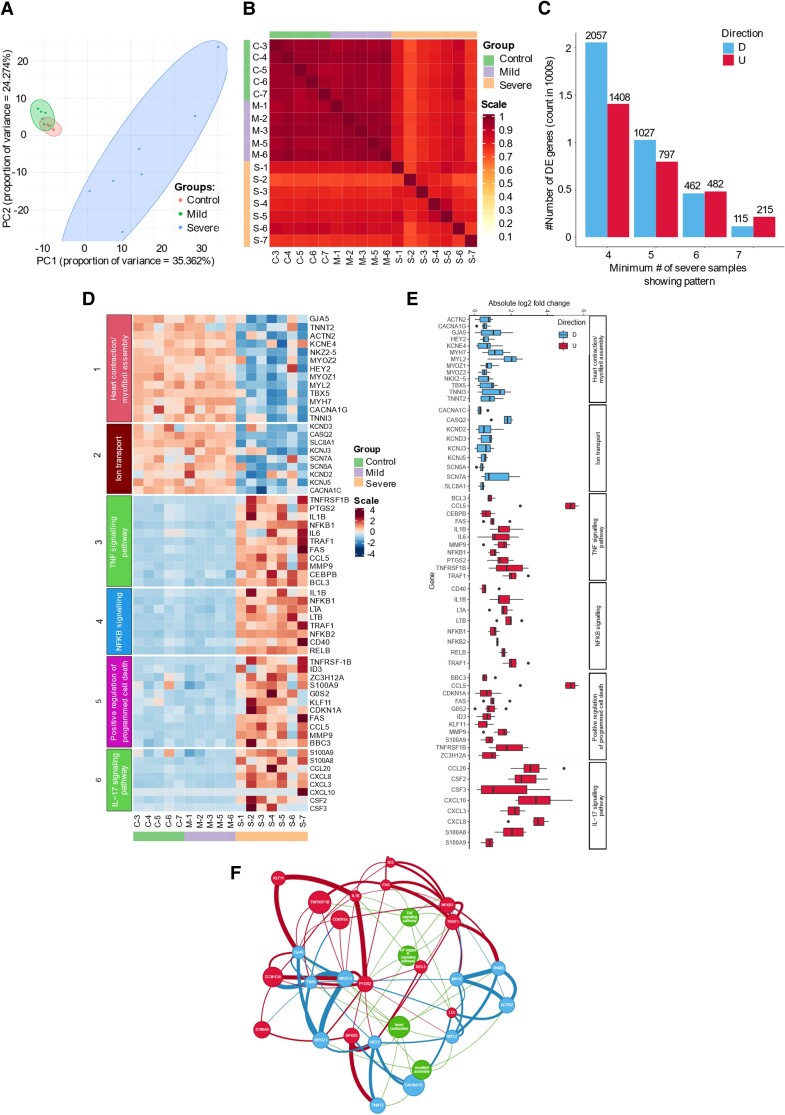
RNA sequencing of serum treated hESC-CM (*A*) principal component analysis of RNA sequencing data calculated on the 500 most abundant genes across all samples of hESC-CM treated with control (*n* = 5, red), mild (*n* = 5, green), or severe (*n* = 7, blue) serum. (*B*) Pairwise JSI, calculated on the 500 most abundant genes across all samples. Ranging from 0 to 1, with high values corresponding to increased similarity. (*C*) Bar chart summarizing the number of genes with log2 (FC) > 0.5 (‘U’, up) or log2 (FC) < −0.5 (‘D’, down) consistently observed when comparing 4/5/6/7 (out of 7) severe samples against all mild/control samples (non-severe); each individual severe sample was compared independently using edgeR. (*D*) Heatmaps showing Z-scores for quantile-normalized expression levels for selected (differentially expressed) genes grouped according to relevant GO or KEGG pathway terms. (*E*) Box plots of distributions of |log2 (FC)| for selected genes across the seven comparisons between all mild/control samples and each severe sample, using edgeR. Genes are grouped according to annotated GO or KEGG pathway terms. The colour reflects upregulation (red) or downregulation (blue) in the severe samples. (*F*) GENIE3 inferred GRNs focused on selected differentially expressed genes. Blue and red nodes correspond to DE genes; the colours reflect downregulation or upregulation, respectively, in severe samples. The width of connecting edges is proportional to weight from the GENIE3 adjacency matrix. Green nodes indicate GO and KEGG pathway terms linked to the DE genes.

To study the gene expression landscape of cardiomyocytes following exposure to sera, we performed bulk RNA sequencing on cells treated with sera from control, mild and severe COVID-19 patients. PCA (Principal component analysis) and Jaccard similarity index (JSI) summaries show that samples exposed to serum coming from severe COVID-19 patients cluster separately from samples exposed to control or mild COVID-19 sera (*Figure 5A* and *B*). Since cardiomyocytes treated with mild COVID-19 or control sera behave similarly and have highly similar gene expression profiles, differential expression analysis was performed between severe and control/mild groups separately and comparing severe against a combined non-severe group. We then specifically looked at genes that were significantly up or downregulated in the severe vs. non-severe cohorts; the differential expression was assessed per severe sample against the non-severe cohort. Given the variability in gene expression observed within the severe group and to avoid the loss of information we systematically looked at genes (and corresponding pathways) that were significantly up or downregulated in at least 5 out of 7 samples (*Figure 5C*).

GSEA (Gene set enrichment analysis), performed using gProfiler shows that within the top upregulated pathways in the severe group several gene ontology (GO), KEGG, and Reactome terms associated with inflammation including cytokine-cytokine receptor interaction, chemokine-mediated signalling pathway and positive regulation of inflammatory response as well as terms associated with apoptosis such as cell death, positive regulation of programmed cell death and positive regulation of apoptotic process can be found (*Figure 5D* and *E*, see Supplementary material online, file  *S1*–S2).

Of interest, TNF signalling pathway was amongst the top enriched terms related to inflammation with genes encoding for pro-inflammatory cytokines such as IL-6, IL1-β, and CCL5 being significantly upregulated by cardiomyocytes treated with serum from severe patients. This finding was in agreement with our cytokine blocking experiment, confirming the role of TNF-α as a main driver of the serum driven phenotype observed in cardiomyocytes and suggested that the inflammatory signal triggered by TNF-α in the serum is then amplified further by increased cytokine expression. Of note, the NFkB (Nuclear factor kappa-light-chain-enhancer of activated B cells) pathway was also significantly upregulated following COVID-19 serum exposure indicating that, at least in part, the signalling cascade was driven via this route (*Figure 5D* and *E*). Our analysis also revealed the IL-17 signalling cascade to be amongst the significantly upregulated KEGG terms. This is of particular interest as IL-17 has been shown to contribute to cardiomyocyte apoptosis in ischemia/reperfusion models and it is known to induce the expression of alarmins (S100A8, S100A9; *Figure 5D* and *E*) which further contribute to the overall inflammatory environment and cardiomyocyte damage.^41–43^

Consistent with our functional data showing decreased contractility, terms associated with heart contraction such as cardiac cell development, regulation of heart contraction, cardiac muscle cell differentiation, myofibril assembly, and ion transport were amongst the significantly downregulated pathways with expression of genes encoding for sarcomeric proteins such as MYH7 and TNNI3, key cardiac transcription factors such as NKX2.5 and HEY2 and ion channels such as SCN5A and SLC8A1 being significantly decreased in most of the samples treated with the severe COVID-19 sera (*Figure 5D* and *E*). RT-qPCR was also performed for a subset of genes to confirm the RNA sequencing results (see Supplementary material online, *Figure S10A*). These conclusions all hold in the severe vs. non-severe comparison as well as the individual severe vs. mild and severe vs. control comparisons (see Supplementary material online, *Figure S10B* and *C*).

We analysed the gene expression profile of cardiomyocytes treated with ARDS vs. control serum. There was a large gene expression variability in the ARDS samples possibly reflecting the different aetiology of this disease (see Supplementary material online, *Figure S11A* and *B*), we however observed how overall expression of genes relates to cardiac development and contractions were downregulated whilst genes associated with an inflammatory response were upregulated in a similar fashion to what we observed in cells treated with severe COVID-19 sera (see Supplementary material online, *Figure S11C*).

Finally, we inferred gene regulatory networks, based on co-variation, on selected differentially expressed genes implicated in the pathways described above using GENIE3. Importantly, *Figure 5F* shows a high degree of connections and co-variation between upregulated genes linked to TNF-α and NFkB pathways (shown in red) and downregulated genes associated with contractility sarcomere assembly (shown in blue) confirming that the activation of inflammatory pathways, at least in part via TNF-α is directly involved in the loss of contractility observed in cardiomyocytes treated with severe COVID-19 sera.

## 4. Discussion

Cardiovascular involvement and associated complications are common in COVID-19 and are associated with a poor disease trajectory. In particular, there has been evidence that SARS-CoV-2 can cause infection and direct viral cytotoxic damage of human myocardium. *In vitro* it has been demonstrated that SARS-CoV-2 can infect hESC-derived cardiomyocytes, significantly impairing cellular structure and function, ultimately resulting in cell death.^4–6^ Adult human myocardium is equipped with the necessary accessory proteins to uptake SARS-CoV-2 and there is sparse evidence from post-mortem studies that an infection is possible in principle.^7,9,10^ Despite this line of evidence, viral myocarditis as a complication of COVID-19 is rarely encountered in clinical practice, indicating that other mechanisms dominate the cardiovascular disease process. Indeed, hyperinflammation is a hallmark of COVID-19,^11,44^ however, serological mechanisms remain a poorly investigated cause of cardiovascular involvement.

Here we demonstrate marked effects of COVID-19 patient sera on hESC-derived cardiomyocytes, significantly impairing cell viability, structure, and function. Our bioassay provides novel mechanistic insight into the serological drivers of cardiotoxicity and the activated downstream pathways in human cardiomyocytes in SARS-CoV-2 and highlights points of action for therapeutic intervention. It also substantiates the clinical observation that male gender, older age, and positive Troponin on serological testing result in a more severe disease trajectory.

Importantly, elucidation of putative serum mediators revealed a significant upregulation of key cytokines in COVID-19 positive patients compared to controls, including IL-6, TNF-α, IL-1β, IL-10, and CRP. While it has previously been demonstrated that serum components result in cardiotoxicity, Mills et al mainly examined a synthetic combination of cytokines, and only examined serum from six patients.^45^ In contrast, we used patient serum from 39 patients and 12 healthy controls, thus showing for the first time a direct link between clinical severity, patient serology, cardiomyocyte (CM) toxicity and activated downstream effectors, an effect that was cell line independent.

The observation that COVID+ but Troponin- serum resulted in a significant reduction of cardiomyocyte viability could be explained by a greater sensitivity of our bioassay than conventional Troponin testing used in routine clinical practice. In the human heart cardiomyocytes are tightly embedded in a 3D-environment of extracellular matrix and neighbouring cells where exposure to cytotoxic serum components potentially does not impact viability as immediately as seen in our bioassay using monolayer sheets of hESC-CMs. An additional explanation includes a potentially missed troponin rise after blood samples were obtained. Spiking of COVID-19 serum with commercially available cytokines failed to impair cardiomyocyte viability suggesting that other drivers, likely acting in synergy, are required for this. This finding is corroborated by the fact that addition of IL-6- and TNF-α-blocking antibodies only resulted in a partial cardiomyocyte rescue suggesting a contributory role of other components.

Our results also highlight the fact that serum driven cardiotoxicity is not specific to COVID-19 as serum from ARDS patients resulted in a similar phenotype to the one observed with severe COVID-19 patients with no significant differences between diseases. Nevertheless, this should not detract from the fact that serum-induced cardiotoxicity remains a largely understudied and poorly understood phenomenon. At the same time a better mechanistic understanding could substantially benefit therapeutic applications for patients.

To elucidate pathways and mediators of the serum-driven toxicity in cardiomyocytes we performed bulk RNA sequencing. Our results show upregulation of pro-apoptotic genes as well as key proinflammatory mediators such as CCL5, IL-6 and IL1β which have been demonstrated to orchestrate the recruitment of several inflammatory cell subsets within the myocardium which might amplify the inflammatory status and increase tissue damage.^46^ Enrichment analysis revealed that, in line with clinical serum analysis, the TNF signalling cascade is amongst the top upregulated pathways.

Our enrichment data indicate that signalling via the NF-kB pathway is a predominant feature in the response to COVID-19 sera. In this context, it has previously been shown that prolonged activation of NF-κB promotes heart failure by eliciting signals that trigger chronic inflammation leading to endoplasmic reticulum stress responses and cell death.^47^ Furthermore, we also observed an upregulation of genes associated with IL-17 signalling pathway. IL-17 has been previously shown to be amongst the cytokines increased in COVID-19 patients and to induce cardiomyocyte apoptosis.^41–43^ Due to the low volumes of serum we had at our disposal we were unable to perform IL-17 ELISA on our samples but we theorize that IL-17 is potentially another cytokine mediator that affects cardiomyocytes.

These changes are paralleled by a downregulation of pathways associated with cardiac muscle contraction, as seen by a decrease in expression of the key cardiac transcription factors Hey2 and Nkx2.5. Similarly, sepsis results in a downregulation of cardiac mitochondrial and sarcomeric genes.^48^ Our bioassay provides an open platform to explore this mechanism in greater depth, signposting the way for future studies.

The importance of inflammatory cytokines such as TNF-α and IL-6 is further demonstrated by the fact that blockade of individual cytokines resulted in a partial rescue of cardiomyocyte viability, confirming serological cytokine-driven cardiotoxicity in COVID-19. This is of particular interest as clinical trials, including REMAP-CAP demonstrated improved outcomes and survival in patients treated with IL-6 antagonists, whereas others such as COVACTA were less conclusive.^49,50^ The fact that combinatorial blockade resulted in significant cardiomyocyte rescue in our study, warrants studying this in a clinical trial and raises the question whether patients severely ill with COVID-19 and a myocardial injury should be treated more aggressively.

Finally, since the occurrence of microthrombi is an integral part of the clinical picture seen in patients severely sick with COVID-19 and the hyperinflammatory disease state constitutes a key mediator in this process^51,52^ we studied the effects of serum treatment on endothelial cells. Our results demonstrate that COVID-19 driven cytotoxicity is cell selective not resulting in impaired endothelial cell viability but resulting in cellular activation and upregulation of ICAM-1 with subsequent immune cell adhesion and vWF deposition in the extracellular matrix. This is an important finding that provides a mechanistic basis for the prothrombogenic disease state seen in patients.^53^

While clinical evidence suggests the use of therapeutic anticoagulation in patients with moderate disease,^54–56^ therapeutic blockade of serological proinflammatory mediators to prevent cardiotoxic damage and an associated adverse disease trajectory early on, is likely to also have beneficial effects on the procoagulant state in COVID-19.

## 5. Strengths and limitations

A strength of our assay is the robust correlation of cardiomyocyte viability with clinical disease severity. Patients requiring supplemental oxygen or assisted ventilation demonstrated significantly lower levels of cardiomyocyte viability in the bioassay and higher levels of proinflammatory serum cytokines than patients with a mild disease course or healthy controls. This was further corroborated by the fact that the presence of higher levels of cytokines accounted for a negative correlation with cardiomyocyte viability and higher levels of proinflammatory cytokines clustered with patients of greater disease burden.

Furthermore, this is the first study to mechanistically investigate the downstream pathways of COVID-19 serum components in human cardiomyocytes comparing them to ARDS. It is not surprising that cardiomyocyte viability and contractility were also impaired by ARDS components. RNA sequencing also shows downstream signalling pathways in human cardiomyocytes are also shared between these two conditions. Our results suggest that serum hyperinflammation causes endothelial activation resulting in immune cell extravasation and thrombogenesis which in turn might amplify myocardial inflammation and contribute to cardiomyocyte death.^57^

A key limitation of our study is that our *in vitro* model might not necessarily recapitulate in full the events occurring *in vivo* where cardiomyocytes are not directly exposed to serum while endothelial cells are. It is important to highlight that this study was neither designed to investigate direct viral cytotoxicity nor the effect of other immune cells on cardiomyocyte viability.

Overall, this study deepens our mechanistic understanding of the cytotoxic damage inflicted on cardiomyocytes in hyperinflammatory states and might provide therapeutic points of action to avert myocardial damage and eventually ameliorate disease burden and prognosis.

## 6. Conclusions

In this study we demonstrate that serum from patients suffering from COVID-19 results in marked cardiotoxicity, an effect increasing with disease severity. We identify key proinflammatory cytokines as drivers of this effect demonstrating greater expression in more severe disease and provide a detailed characterization of the downstream pathways activated in human cardiomyocytes. The effects of serum-induced cytotoxicity are cell specific and do not affect microvascular endothelial cells. Combinatorial blockade of IL-6 and TNF-α results in a significant increase of cardiomyocyte viability, indicating an opportunity for therapeutic intervention in severe COVID-19.

## Supplementary material

Supplementary material is available at *Cardiovascular Research* online.

## Supplementary Material

cvad174_Supplementary_Data

## Data Availability

The raw data supporting the findings of this study are available from the corresponding author upon reasonable request.

## Notes

Translational perspective Cardiovascular complications are common in patients with COVID-19 and worsen the clinical disease trajectory. While pre-clinical studies have demonstrated that SARS-CoV-2 can infect and replicate within human stem cell-derived cardiomyocytes, clinical evidence of this mechanism leading to a viral myocarditis remains sparse. At the same time the effects of the proinflammatory cytokine response in COVID-19 on the heart remain largely unexplored. Here we show that serum from patients with confirmed COVID-19 impairs the viability of hESC-derived cardiomyocytes and identify key proinflammatory mediators of this effect, which is alleviated by therapeutic blockade. Furthermore, we demonstrate that serum exposure results in activation of microvascular endothelial cells and a procoagulant state, providing important mechanistic context of the clinical observation of pro-coagulopathy in COVID-19. This study demonstrates that serum components are likely to drive cardiotoxicity in COVID-19 and highlights targets for therapeutic intervention.
