# Supplementary material for: Proinflammatory cytokines driving cardiotoxicity in COVID-19
Source: Cardiovasc Res. 2023 Dec 2;120(2):174–87. doi: 10.1093/cvr/cvad174 (PMC10936751; doi:10.1093/cvr/cvad174)
Supplement: cvad174_Supplementary_Data [file cvad174_supplementary_data.docx]

**Proinflammatory cytokines driving cardiotoxicity in Covid-19**

Maria Colzani^1,2^*, Johannes Bargehr^1,2^*, Federica Mescia^2,3^, Eleanor Williams^1^, Vincent Knight-Schrijver^1,2^, Jonathan Lee ^1,2^, Cambridge Institute of Therapeutic Immunology and Infectious Disease-National Institute of Health Research (CITIID-NIHR) COVID BioResource Collaboration, Charlotte Summers^2,4^, Irina Mohorianu^1^, Kenneth G C Smith^2,3^, Paul A Lyons^2,3^, Sanjay Sinha^1,2^

*These authors share first authorship.

**Affiliations:**

^1^ Wellcome – MRC Cambridge Stem Cell Institute, Jeffrey Cheah Biomedical Centre, Cambridge Biomedical Campus, University of Cambridge, Puddicombe Way, CB2 0AW Cambridge, UK

^2^ Department of Medicine, School of Clinical Medicine, University of Cambridge, Cambridge Biomedical Campus, CB2 0SP Cambridge, UK.

^3^ Cambridge Institute of Therapeutic Immunology and Infectious Disease, Jeffrey Cheah Biomedical Centre, Cambridge Biomedical Campus, CB2 0AW Cambridge, UK.

^4^ Wolfson Lung Injury Unit, Heart and Lung Research Institute, Papworth Road, Cambridge Biomedical Campus, CB2 0BB

**Running title: Cardiotoxicity in COVID-19**

**Corresponding author:**

Sanjay Sinha (ss661@cam.ac.uk)

Wellcome – MRC Cambridge Stem Cell Institute

Jeffrey Cheah Biomedical Centre

Cambridge Biomedical Campus

University of Cambridge

Puddicombe Way

CB2 0AW Cambridge

UK

**Supplementary material online:**

**Figure S1. Cardiac Differentiation protocol schematic**


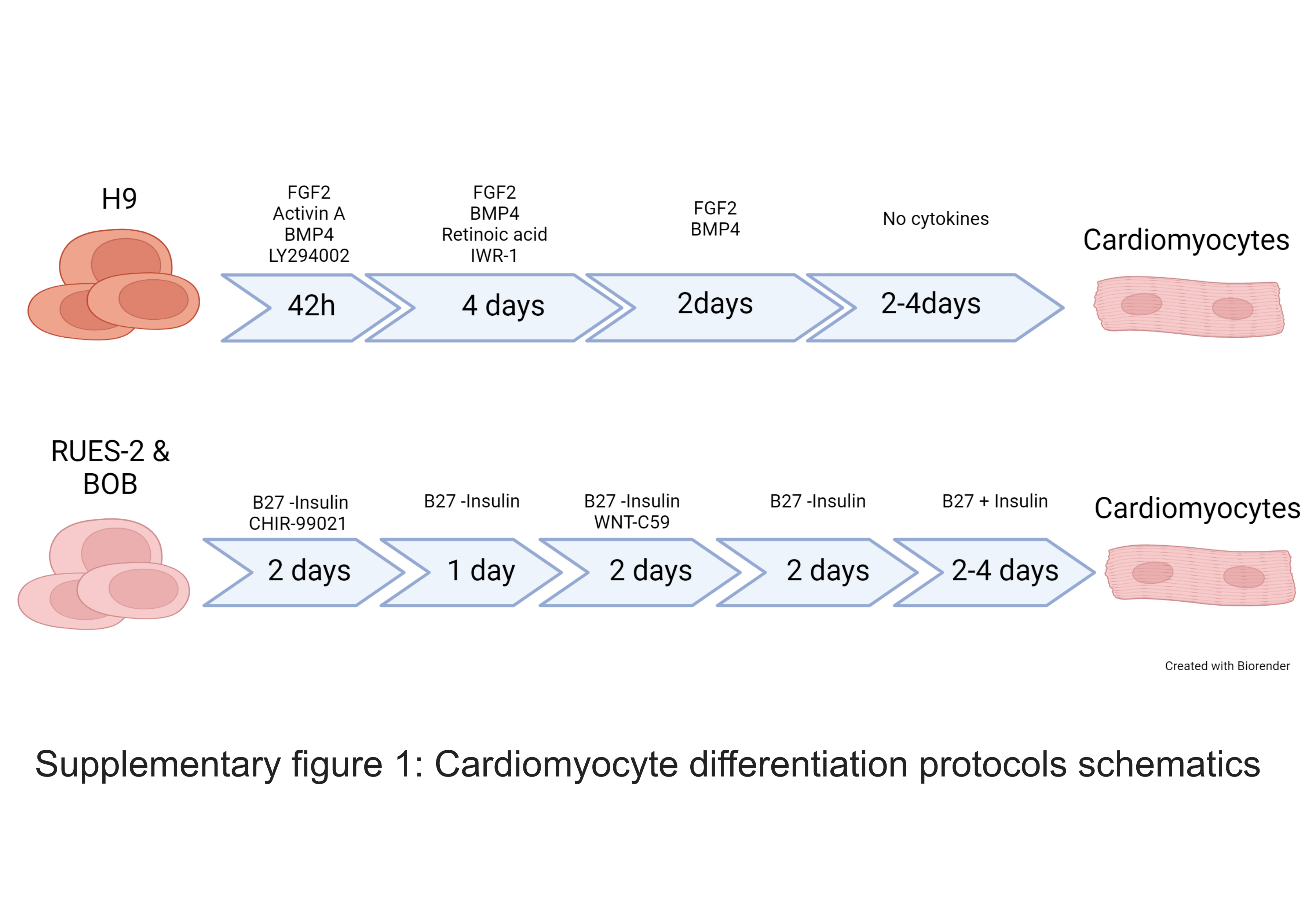


**Figure S1:** Schematics of the cardiac differentiation protocols. The schematic depicts directed differentiation of H9-hESC and RUES-2 as well as BOB hiPSC lines to cardiomyocytes. **TOP:** H9 hESCs and BOBC hiPSC were plated in Matrigel (Corning)- coated 12 well plates at a seeding density of 105 cells/cm2 in CDM-BSA supplemented with ROCK inhibitor (Millipore, 1 μm). After 3 hours the media was changed to CDM-BSA supplemented with FGF-2 (20 ng/ml), Activin-A (50 ng/ml), BMP-4 (10 ng/ml, R&D) and LY294002 (10 μM Tocris) (FLyAB media). Following a 42-hour culture period, the media was changed to CDM-BSA containing FGF-2 (8 ng/ml), BMP4 (20 ng/ml), Retinoic Acid (SIMGA, 1 μM), endo-IWRI (1 μM, TOCRIS) (FBRI media) and cells maintained in this medium for 4 days with media change every 48hrs. Subsequently, the media was changed to CDM-BSA containing FGF-2 (8 ng/ml), BMP4 (20 ng/ml) for an additional 2 days. Cells were then cultured with CDM-BSA with no cytokines with media changes every 48hrs (see Supplementary material online, Figure S1). **BOTTOM:** RUES2 hESC were plated in a 6 well plate at a density of 2.5 x 10^6^ million cells per well prior to incubation with CHIR-99021 (6µM, Tocris) for 2 days in RPMI media supplemented with B27 supplement without insulin followed by a 1-day washout period. This was followed by a 48-hour incubation with RPMI-B27 minus insulin supplemented with Wnt-C59 (2.5 µM, Stratech). After 48 hours media was replaced with RPMI-B27 with insulin and refreshed every 2 days.

**
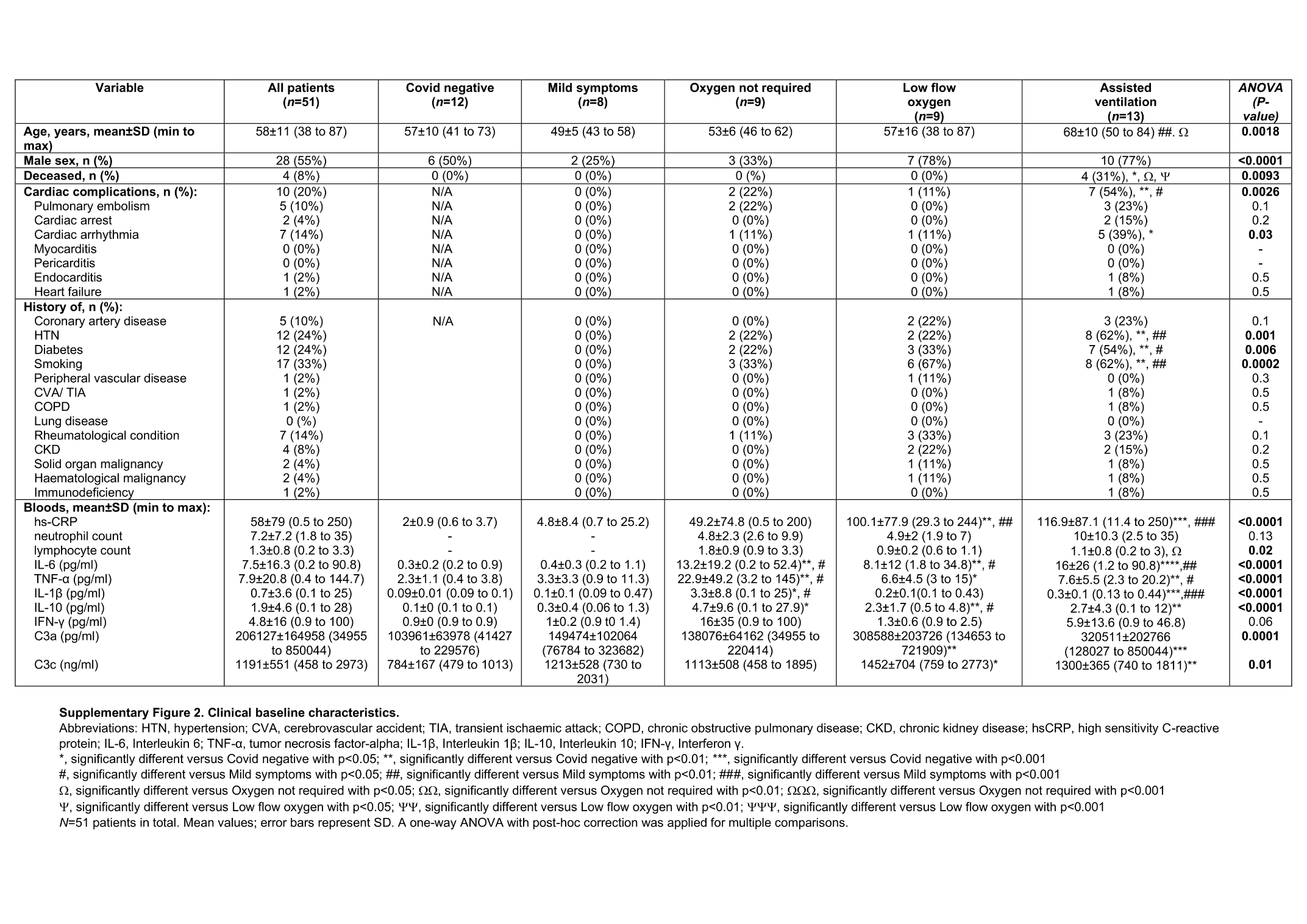
Figure S2. Clinical baseline characteristics.**

**Figure S2. Clinical baseline characteristics.**

Abbreviations: HTN, hypertension; CVA, cerebrovascular accident; TIA, transient ischaemic attack; COPD, chronic obstructive pulmonary disease; CKD, chronic kidney disease; hsCRP, high sensitivity C-reactive protein; IL-6, Interleukin 6; TNF-α, tumor necrosis factor-alpha; IL-1β, Interleukin 1β; IL-10, Interleukin 10; IFN-γ, Interferon γ.

*, significantly different versus Covid negative with *p*<0.05; **, significantly different versus Covid negative with *p*<0.01; ***, significantly different versus Covid negative with *p*<0.001.

#, significantly different versus Mild symptoms with *p*<0.05; ##, significantly different versus Mild symptoms with p<0.01; ###, significantly different versus Mild symptoms with *p*<0.001

Ω, significantly different versus Oxygen not required with *p*<0.05; ΩΩ, significantly different versus Oxygen not required with *p*<0.01; ΩΩΩ, significantly different versus Oxygen not required with *p*<0.001.

Ψ, significantly different versus Oxygen-non assisted with p<0.05; ΨΨ, significantly different versus Oxygen-non assisted with *p*<0.01; ΨΨΨ, significantly different versus Oxygen-non assisted with *p*<0.001.

*N*=51 patients in total. Mean values; error bars represent SD. A one-way ANOVA with post-hoc correction was applied for multiple comparisons.

**Figure S3. Ca^2+^-imaging and cardiac Troponin-T ELISA of hESC-CM following serum exposure.**

**
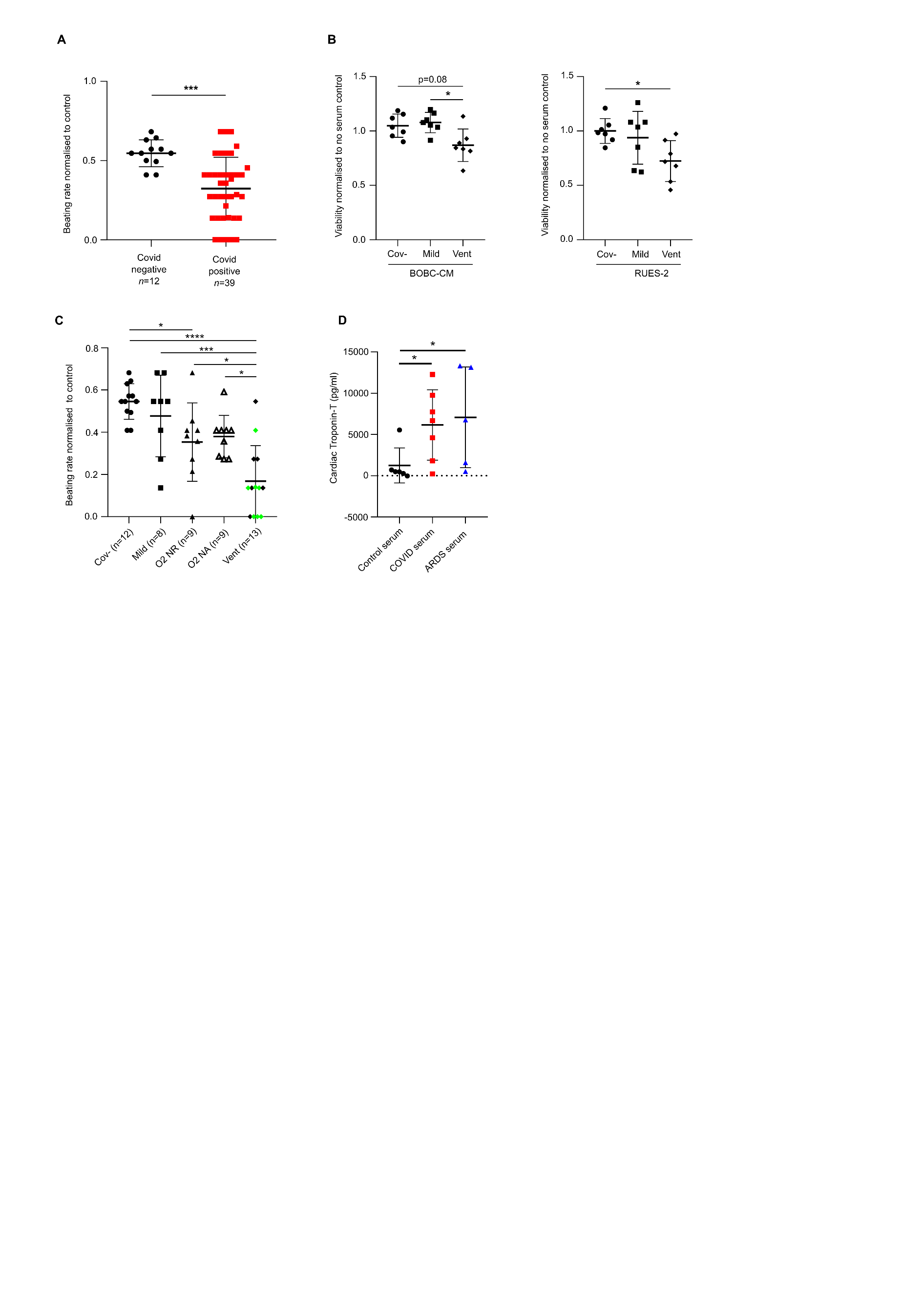
**

**Figure S3. Ca2+-imaging and cardiac Troponin-T ELISA of hESC-CM following serum exposure. (A)** HESC-CM beating rate following exposure to patient serum normalised to no serum control. Covid negative, covid positive, *n*=12 and 39. **(B)** Worsening serum cardiotoxicity with increasing clinical disease severity demonstrated with different hPSC lines, BOBC (male) and RUES-2 (female). **(C)** hESC-CM beating rate stratified according to disease severity. Cov-, mild, O2 NR, O2 NA, vent, *n*=12, 8, 9, 9 and 13. **(D)** Results of cardiac troponin T ELISA assay. Cardiac Troponin-T release in the cell culture supernatant measured by ELISA in cardiomyocytes treated with Control (*n*=6) Severe COVID-19 (*n*=7) and ARDS (*n*=5) sera. Serum containing media not exposed to the cells was used as a control to ensure that cardiac Troponin-T detected in the cell culture supernatant was of cardiomyocyte origin.

Mean values; error bars represent s.d. Two-sided P values were calculated using an unpaired T-Test for (A) and using a one-way ANOVA with post-hoc correction for multiple comparisons (B). Abbreviations: Cov-, COVID-19 negative; mild, mild symptoms only; O2 NR, oxygen not required; O2 NA, Oxygen-non assisted; vent, assisted ventilation; hESC-CM, human embryonic stem cell-derived cardiomyocytes.

**Figure S4. Neutrophil, lymphocyte counts and cardiac complications.**


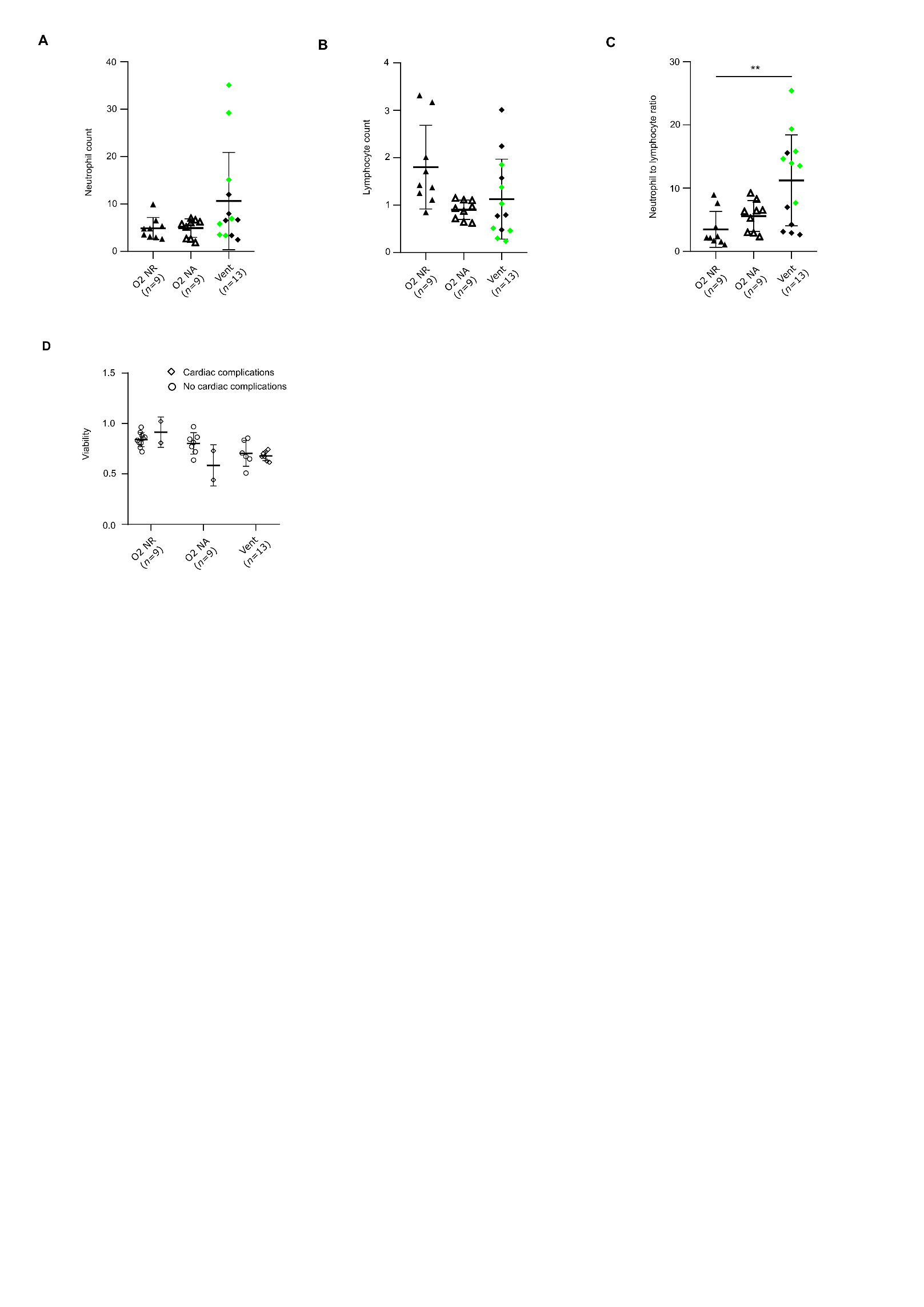


**Figure S4. Neutrophil, lymphocyte counts and cardiac complications. (A)** Neutrophil count in patients with confirmed COVID-19, requiring no oxygen, Oxygen-non assisted or assisted ventilation. **(B)** Lymphocyte count inpatients with confirmed COVID-19. **(C)** Neutrophil to lymphocyte ratio in patients with confirmed COVID-19. **(D)** Cardiac complications in patients with confirmed COVID-19, requiring no oxygen, Oxygen-non assisted, or assisted ventilation. O2 NR, O2 NA, Vent, *n*=9, 9 and 13. Mean values; error bars represent s.d. Two-sided P values were calculated using a one-way ANOVA with post-hoc correction for multiple comparisons. ** *P*< 0.01. Abbreviations: Cov-, COVID-19 negative; mild, mild symptoms only; O2 NR, oxygen not required; O2 NA, Oxygen-non assisted; vent, assisted ventilation; hESC-CM, human embryonic stem cell-derived cardiomyocytes.

**Figure S5. Serum cardiotoxicity in cohort 1.**


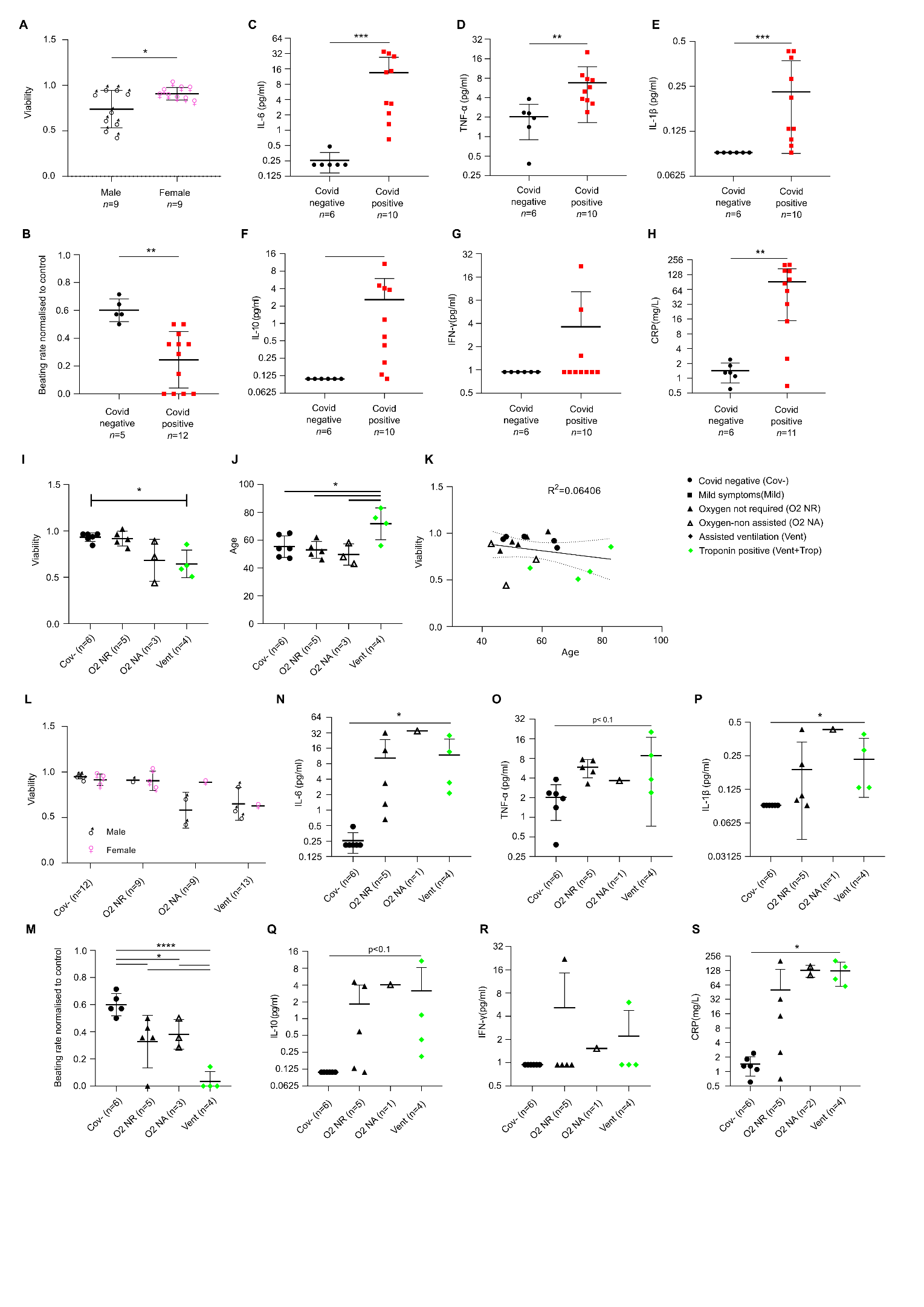


**Figure S5. Serum cardiotoxicity in cohort 1. (A)** Gender distribution among patients of cohort 1. **(B)** HESC-CM beating rate normalised to no serum control following exposure to patient serum normalised to control group. **(C-H)** ELISA-multiplex assay of patient serum samples, including IL-6 **(C)**, TNF-α **(D)**, IL1-β **(E)**, IL-10 **(F)**, IFN-γ **(G)** and CRP **(H)**. **(I)** hESC-CM viability normalised to no serum control stratified according to disease severity. **(J)** Age in patients stratified according to disease severity. **(K)** Correlation of age and hESC-CM viability. **(L)** Gender distribution stratified according to disease severity. **(M)** HESC-CM beating rate normalised to no serum control stratified according to disease severity. **(N-S)** ELISA-multiplex assay results in patients stratified according to disease severity, including IL-6 **(N)**, TNF-α **(O)**, IL1-β **(P)**, IL-10 **(Q)**, IFN-γ **(R)** and CRP **(S)**.

Mean values; error bars represent s.d. Two-sided P values were calculated using an unpaired t-test for **(A-H)** and **(L)** and using one-way ANOVA with post-hoc correction for multiple comparisons for **(I-J)** and **(M-S)**. * *P* < 0.05, ** *P* < 0.01, *** *P* < 0.001, **** *P* < 0.0001. Abbreviations: hESC-CM, human embryonic stem cell-derived cardiomyocytes; Cov-, Covid negative; Mild, mild symptoms; O2 NR, oxygen not required; O2 NA, Oxygen-non assisted; Vent, assisted ventilation; Vent+Trop, troponin positive.

**Figure S6. Serum cardiotoxicity in cohort 2.**


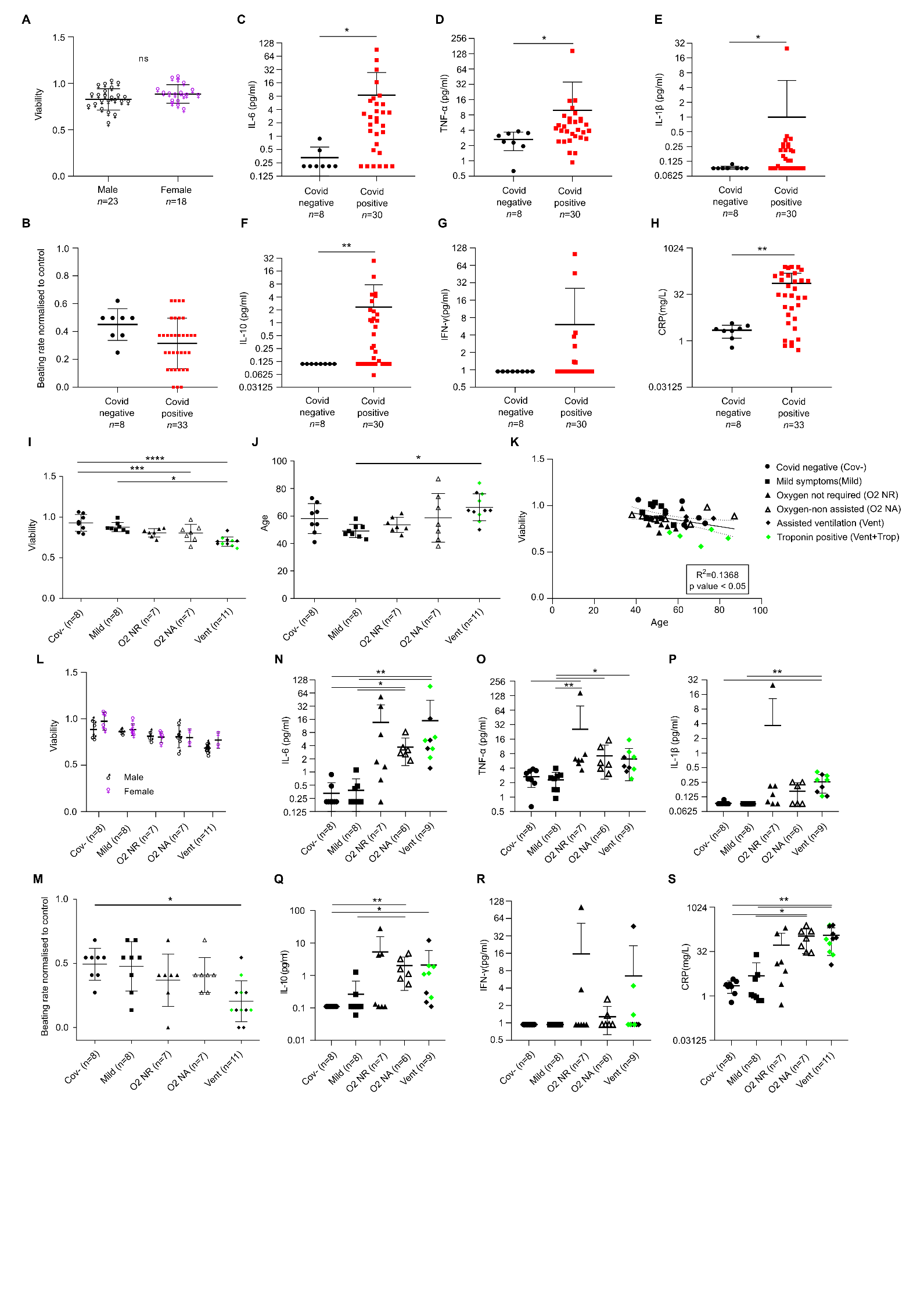


**Figure S6. Serum cardiotoxicity in cohort 2. (A)** Gender distribution among patients of cohort 1. **(B)** HESC-CM beating rate normalized to no serum control following exposure to patient serum normalised to control group. **(C-H)** ELISA-multiplex assay of patient serum samples, including IL-6 **(C)**, TNF-α **(D)**, IL1-β **(E)**, IL-10 **(F)**, IFN-γ **(G)** and CRP **(H)**. **(I)** hESC-CM viability in patients stratified according to disease severity. **(J)** Age in patients stratified according to disease severity. **(K)** Correlation of age and hESC-CM viability. **(L)** Gender distribution stratified according to disease severity. **(M)** HESC-CM beating rate in patients stratified according to disease severity. **(N-S)** ELISA-multiplex assay results in patients stratified according to disease severity, including IL-6 **(N)**, TNF-α **(O)**, IL1-β **(P)**, IL-10 **(Q)**, IFN-γ **(R)** and CRP **(S)**.

Mean values; error bars represent s.d. Two-sided P values were calculated using an unpaired t-test for **(A-H)** and (**L**) and using one-way ANOVA with post-hoc correction for multiple comparisons for **(I-J)** and **(M-S)**. * *P* < 0.05, ** *P* < 0.01, *** *P* < 0.001, **** *P* < 0.0001. Abbreviations: hESC-CM, human embryonic stem cell-derived cardiomyocytes; Cov-, Covid negative; Mild, mild symptoms; O2 NR, oxygen not required; O2 NA, Oxygen-non assisted; Vent, assisted ventilation; Vent+Trop, troponin positive.

**Figure S7. Spiking of control serum with commercially available cytokines.**


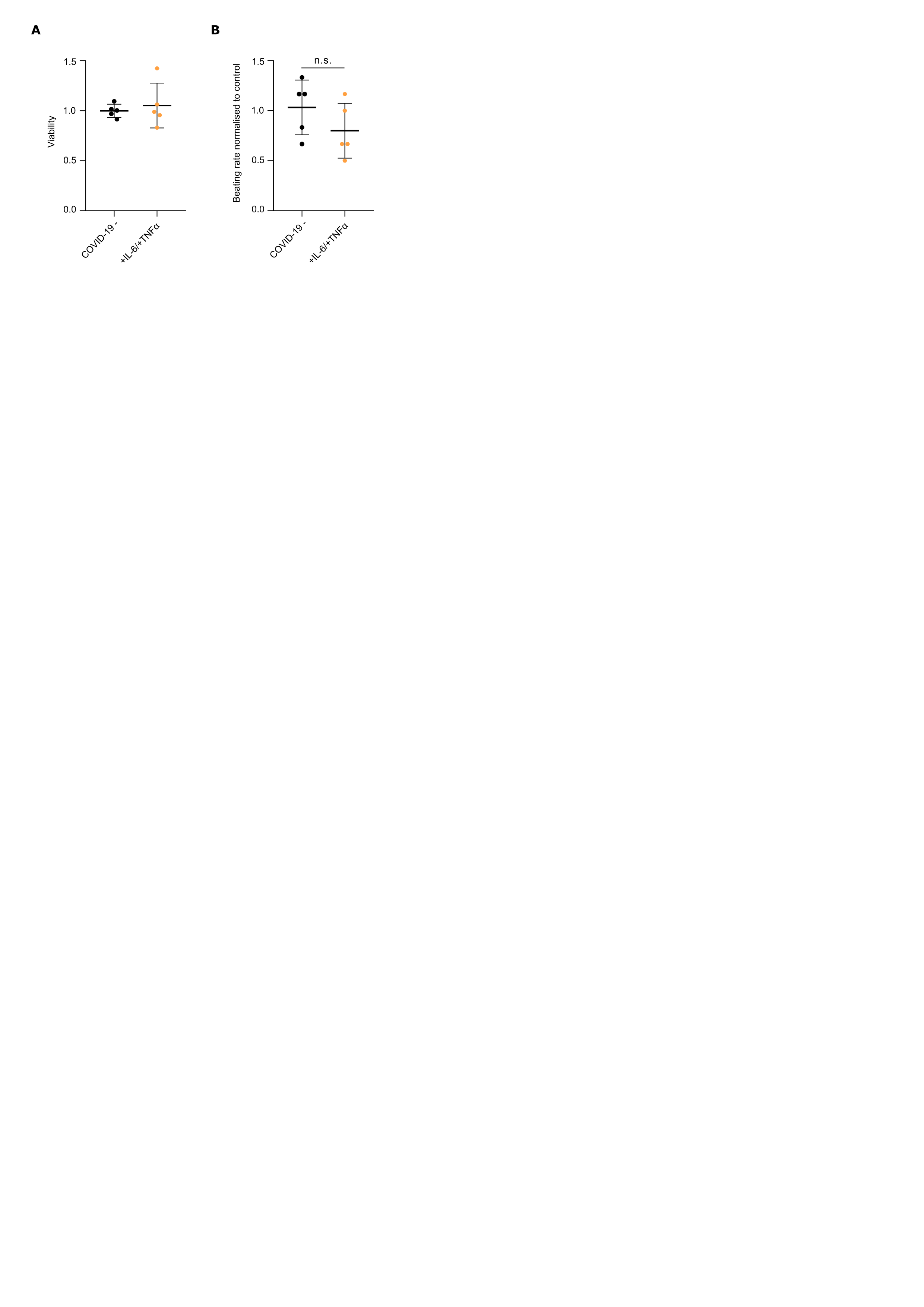


**Figure S7. Spiking of control serum with commercially available cytokines. (A)** Effects of commercially available IL-6 and TNFα on cardiomyocyte viability. **(B)** Effects of commercially available IL-6 and TNFα on beating rate of cardiomyocytes. Abbreviations: n.s.; not significant.

**Figure S8. HESC-CM viability in response to serum four-weeks after detection of COVID-19 infection.**

**
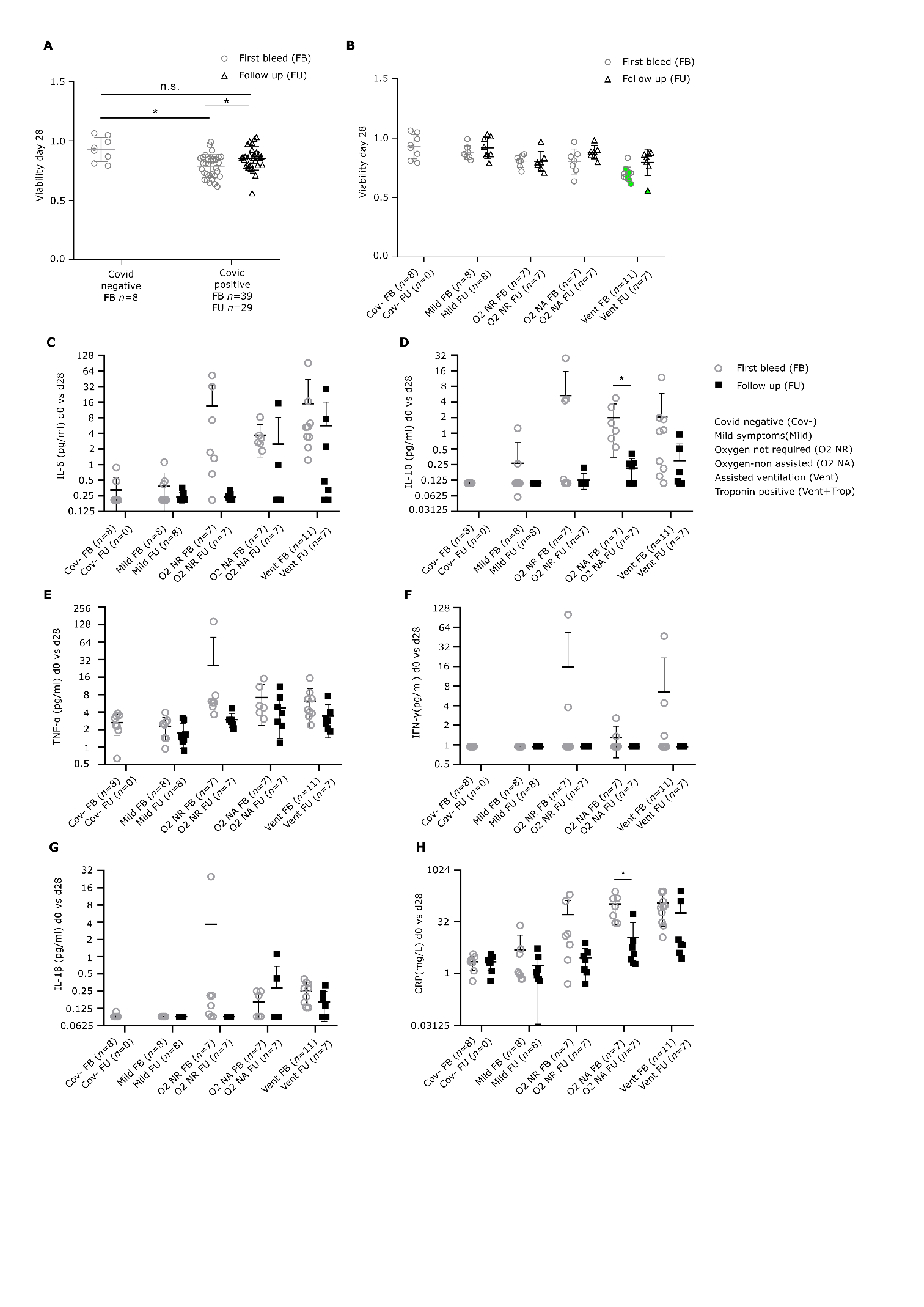
**

**Figure S8. HESC-CM viability in response to serum four-weeks after detection of COVID-19 infection. (A)** hESC-CM viability (normalised to nor serum control) after treatment with serum from the first bleed (FB) or four weeks follow up (FU) in COVID-19 positive patients and negative controls. **(B)** hESC-CM viability amongst the different groups of disease severity. Green symbols denote patients with troponin-positive serum samples. **(C-H)** Cytokine levels on day 0 and day 28 amongst the different groups of disease severity. Time course is shown for IL-6 **(C)**, TNF-α **(D)**, IL1-β **(E)**, IL-10 **(F)**, IFN-γ **(G)** and CRP **(H)**. **(C-H)** Cov- FB, Cov FU, mild FB, mild FU, O2 NR FB, O2 NR FU, O2 NA FB, O2 NA FU, Vent FB, Vent FU, *n*=8, 0, 8, 8, 9, 7, 9, 7, 13 and 7.

Mean values; error bars represent s.d. Two-sided P values were calculated using an unpaired t-test for two groups and using a one-way ANOVA with post-hoc correction for multiple comparisons. * *P* < 0.05. Abbreviations: Cov-, COVID-19 negative; mild, mild symptoms only; O2 NR, oxygen not required; O2 NA, Oxygen-non assisted; vent, assisted ventilation; hESC-CM, human embryonic stem cell-derived cardiomyocytes.

**Figure S9: HL-60 adhesion on serum treated HMVECs.**

**
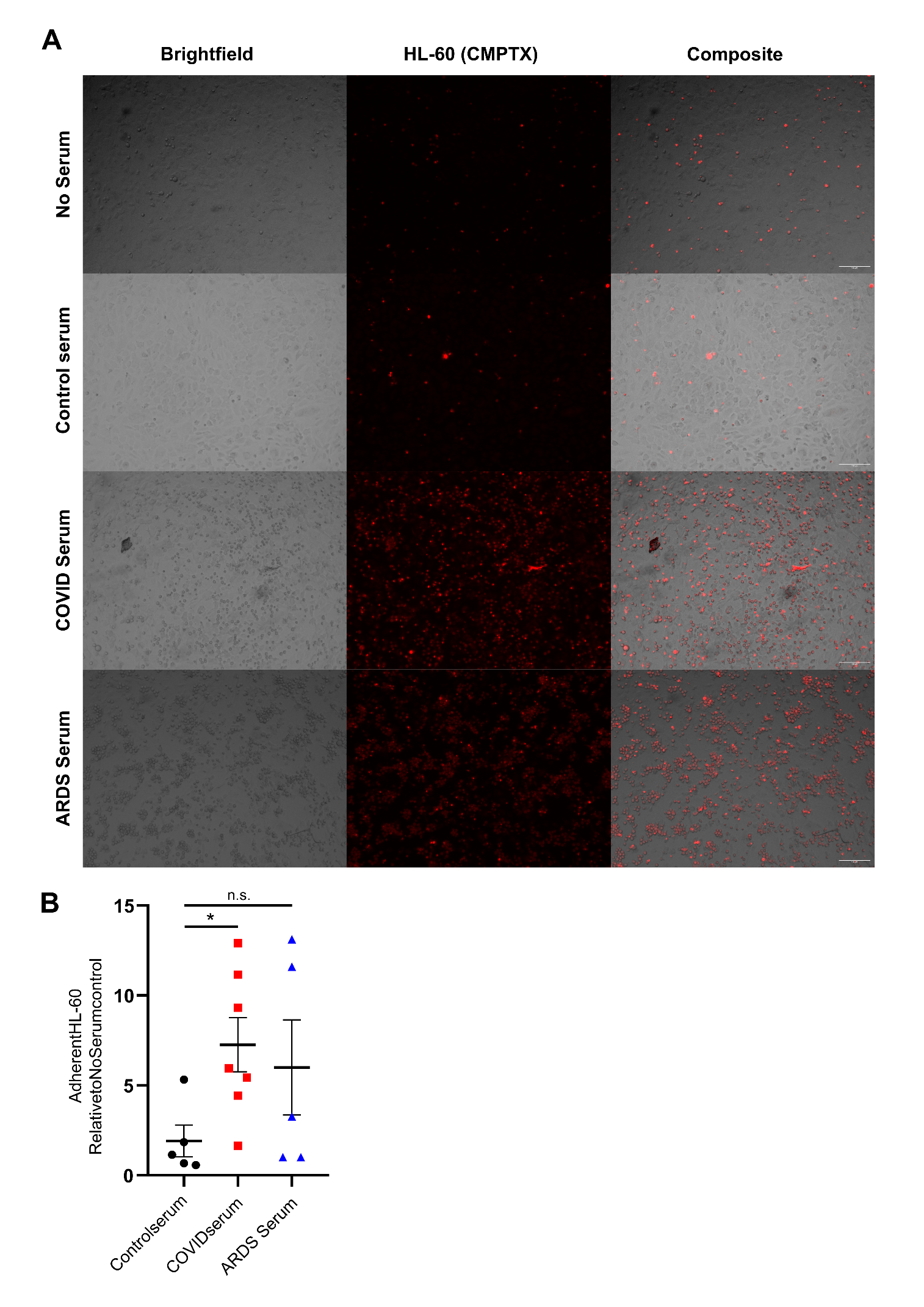
**

**Figure S9: HL-60 adhesion on serum treated HMVECs. (A)** Representative brightfield, CMPTX (red) and composite images of CellTracker red CMPTX labelled HL-60 adhering to HMVECs untreated or treated with Control, Severe COVID and ARDS serum. Scale bar 150 µm **(B)** Quantification of HL-60 adherent cells inControl (*n*=5), Severe COVID (*n*=7) and ARDS serum (*n*=5). The number of adherent cells was normalized to the number of adherent HL-60 on the untreated HMVECs. Mean values; error bars represent s.d. Two-sided P values were calculated using an unpaired t-test for two groups and using a one-way ANOVA with post-hoc correction for multiple comparisons. * *P* < 0.05

**
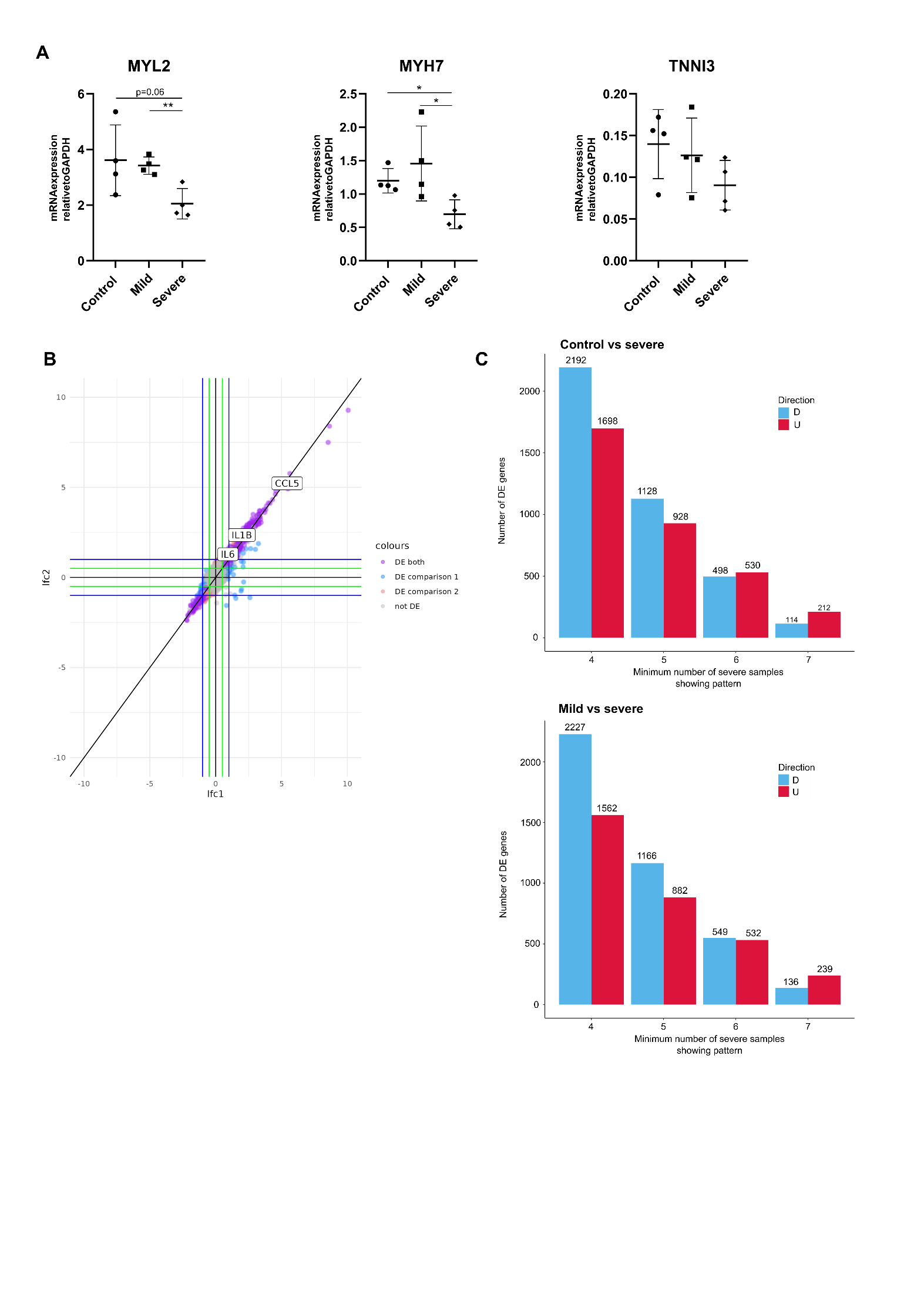
Figure S10. Validation of RNA sequencing analysis**

**Figure S10. Validation of RNA sequencing analysis. (A)** RT-qPCR gene expression analysis of MYL2, MYH7 and TNNI3 relative to GAPDH in hESC derived cardiomyocytes treated with control (*n*=4), mild COVID (*n*=4) and severe COVID serum. **(B)** Cross plot with the log2FC for control vs severe shown on the x-axis and mild vs severe on the y-axis showing high consistency between the two comparisons by following y=x. Key genes are highlighted (CCL5, IL1-b, IL-6) and demonstrate consistent upregulation. **(C)** Bar chart summarising the number of genes with log2 (FC) > 0.5 (‘U’, up) or log2 (FC) < -0.5 (‘D’, down) consistently observed when comparing 4/5/6/7 (out of 7) severe samples against either all control (top graph) or mild samples (bottom graph) respectively, demonstrating consistency in numbers and patterns with comparison of severe against combined mild/control samples as shown in figure 5C; each individual severe sample was compared independently using edgeR.

Mean values; error bars represent s.d. Two-sided *P* values were calculated using an unpaired t-test for two groups and using a one-way ANOVA with post-hoc correction for multiple comparisons. * *P* < 0.05.

**Figure S11. ARDS serum treated hESC-CM RNA sequencing analysis**


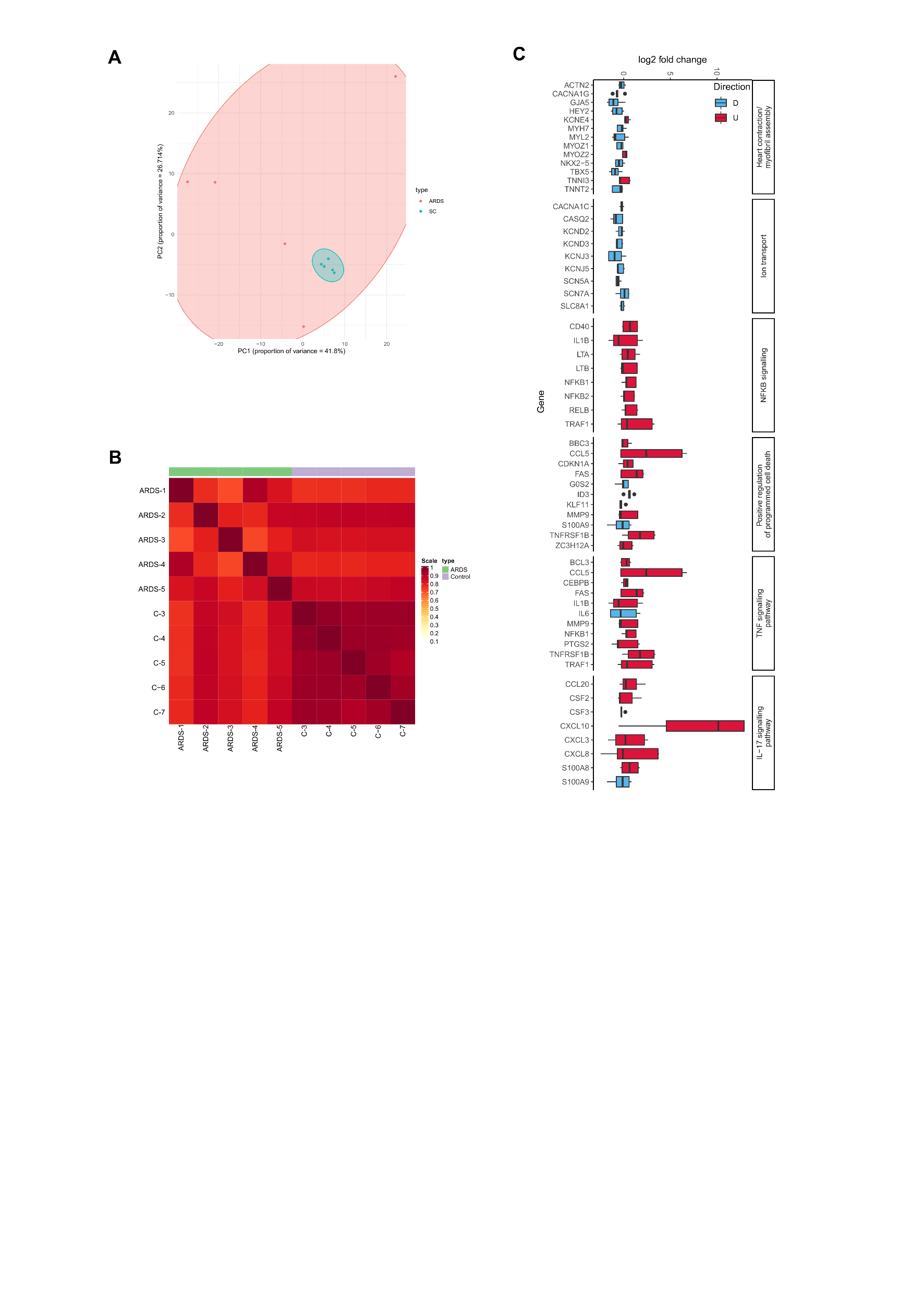


**Figure S11. ARDS serum treated hESC-CM RNA sequencing analysis. (A)** Principal component analysis of RNA sequencing data calculated on the 500 most abundant genes across all samples of hESC-CM treated with Control (*n*=5, green), or ARDS (*n*=5, Red) serum. **(B)** Pairwise Jaccard Similarity Index (JSI), calculated on the 500 most abundant genes across all samples. Ranging from 0 to 1, with high values corresponding to increased similarity. **(C)** Box plots of distributions of log2 (FC) for selected genes across the comparisons between control samples and each ARDS sample, using edgeR. Genes are grouped according to annotated GO or KEGG pathway terms. The colour reflects upregulation (red) or downregulation (blue) in the severe samples.
